# Reduced dynamic loads due to hip dislocation induce acetabular cartilage degeneration by IL-6 and MMP3 via the STAT3/periostin/NF-κB axis

**DOI:** 10.1038/s41598-022-16585-6

**Published:** 2022-07-16

**Authors:** Yutaka Nakamura, Mitsuru Saitou, Shingo Komura, Kazu Matsumoto, Hiroyasu Ogawa, Takaki Miyagawa, Takashi Saitou, Takeshi Imamura, Yuuki Imai, Hiroshi Takayanagi, Haruhiko Akiyama

**Affiliations:** 1 Department of Orthopaedic Surgery, Graduate School of Medicine, Gifu University, 1-1 Yanagido, Gifu, 501-1194 Japan; 2 Department of Molecular Medicine for Pathogenesis, Graduate School of Medicine, Ehime University, Toon, Ehime 791-0295 Japan; 3 Division of Integrative Pathophysiology, Proteo-Science Center, Ehime University, Toon, Ehime 791-0295 Japan; 4 Department of Immunology, Graduate School of Medicine and Faculty of Medicine, The University of Tokyo, Hongo 7-3-1, Bunkyo-ku, Tokyo, 113-0033 Japan

**Keywords:** Cell biology, Molecular biology

## Abstract

Developmental dysplasia of the hip (DDH) is characterized by anatomical abnormalities of the hip joint, ranging from mild acetabular dysplasia to hip subluxation and eventually dislocation. The mechanism underlying the cartilage degeneration of the hip joints exposed to reduced dynamic loads due to hip dislocation remains unknown. We established a rodent hip dislocation (disarticulation; DA) model of DDH (DA-DDH rats and mice) by swaddling. Expression levels of periostin (Postn) and catabolic factors, such as interleukin-6 (IL-6) and matrix metalloproteinase 3 (Mmp3), increased and those of chondrogenic markers decreased in the acetabular cartilage of the DA-DDH models. Postn induced *IL-6* and *Mmp3* expression in chondrocytes through integrin αVβ3, focal adhesion kinase, Src, and nuclear factor-κB (NF-κB) signaling. The microgravity environment created by a random positioning machine induced Postn expression in chondrocytes through signal transducer and activator of transcription 3 (STAT3) signaling. IL-6 stimulated Postn expression via STAT3 signaling. Furthermore, cartilage degeneration was suppressed in the acetabulum of *Postn*^−/−^ DA-DDH mice compared with that in the acetabulum of wild type DA-DDH mice. In summary, reduced dynamic loads due to hip dislocation induced acetabular cartilage degeneration via IL-6 and MMP3 through STAT3/periostin/NF-κB signaling in the rodent DA-DDH models.

## Introduction

The hip joint is composed of the acetabulum and the femoral head, which face each other, and their congruency is required for proper socket-like growth of the acetabulum and maintenance of the articular cartilage^1–4^. Developmental dysplasia of the hip (DDH), also known as developmental dislocation or congenital dislocation of the hip, exhibits anatomical abnormalities of the hip joint, ranging from mild acetabular dysplasia to hip subluxation and eventually dislocation^5,6^. The abnormal contact stress to the acetabulum due to acetabular dysplasia is associated with cartilage degeneration in DDH^7^. Cumulative excessive stress on the articular surface due to joint instability or traumatic damage causes increased wear of the superficial cartilage and leads to osteoarthritis (OA)^8^. Although most of the acetabulum is barely loaded due to hip dislocation since infancy in DDH patients with complete dislocation (International Hip Dysplasia Institute classification grade 3 or 4), cartilage degeneration develops even in unloaded cartilage. A study based on animal models lacking limb musculature or muscle contraction reported the necessity of muscular contraction in forming an appropriate joint cavity and morphogenesis^9^. In addition, mechanical motion protects against cartilage degeneration by promoting *Prg4* expression in articular cartilage^10^. The acetabular dysplasia and acetabular cartilage degeneration occur after hip dislocation in experimental DDH models induced by swaddling^2,3,11,12^. Collectively, appropriate mechanical stress and dynamic loads on the articular cartilage are important for the normal growth of the joint and maintenance of the articular cartilage^9,10^. We hypothesized that the phenotype and mechanism of degeneration in cartilage subjected to reduced dynamic load are different from those of cartilage subjected to excessive stress and traumatic damage.

Periostin (Postn) is an extracellular matrix (ECM) molecule of the fasciclin family, which acts in cell adhesion and migration and plays an important role in maintaining tissue integrity and tissue remodeling^13–15^. Postn is overexpressed in articular chondrocytes and their periphery matrix in human knee OA and rodent models of knee OA^16,17^. Furthermore, Postn loss-of-function suppresses post-traumatic and age-related OA progression^14,18,19^.

In the present study, we created rat hip dislocation (disarticulation; DA) model of DDH (hereafter termed as DA-DDH) and assessed gene expression patterns in their acetabular cartilage. We focused on the relationship between reduced dynamic loads and cartilage degeneration mediated via Postn and analyzed the effects of Postn on articular chondrocytes and the mechanism by which Postn induces expression of catabolic factors in the articular chondrocytes. Additionally, we investigated the intracellular signaling that induced Postn expression in the chondrocytes cultured in the experimental reduced dynamic loaded environment using a random positioning machine (RPM). Finally, we created DA-DDH mice, and assessed cartilage degeneration in the acetabular cartilage of wild type (WT) and *Postn*-knockout (*Postn*^*−/−*^) DA-DDH mice models.

## Results

### Cartilage degeneration in the acetabulum of DA-DDH rats

**Figure 1:**
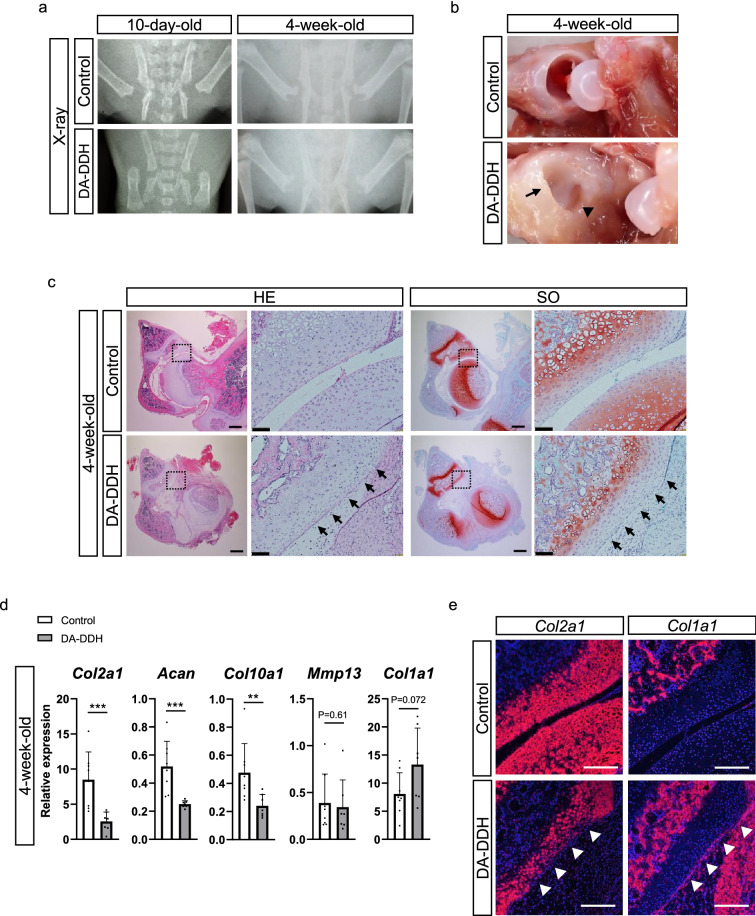
Morphological, histological, and gene expression analyses of the hips disarticulation (DA) in rat models of developmental dysplasia of the hip (DA-DDH). (**a**) Anteroposterior pelvic radiographs of 10-day- and 4-week-old control and DA-DDH rats. (**b**) Images of the hip joints of 4-week-old control and DA-DDH rats. The arrowhead indicates the hypoplastic primary acetabulum, and the arrow indicates the secondary acetabulum in DA-DDH rats. (**c**) Hematoxylin–eosin and safranin O/fast green (SO) staining of the anterior wall of the acetabulum of 4-week-old rats. The dotted squares in the left panel images of each sample are magnified in the right panels. The black arrows indicate cartilage degeneration in the acetabulum. Scale bar: 1 mm, left panel; 50 μm, right panel. (**d**) Relative mRNA expression of *Col2a1*, *Acan*, *Col10a1*, *Mmp13*, and *Col1a1* in the acetabular cartilage of 4-week-old control and DA-DDH rats (n = 8; control, n = 7; DA-DDH). (**e**) In situ hybridization was performed for assessing *Col2a1* and *Col1a1* expression in the acetabulum of 4-week-old control and DA-DDH rats. The white arrowheads indicate downregulated expression of *Col2a1* and upregulated expression of *Col1a1* in the acetabular cartilage. Scale bar, 200 μm. The Mann–Whitney *U* test was used for statistical analysis. Values indicate the mean ± SD. *P < 0.05, **P < 0.01, ***P < 0.001.

We successfully generated a DA-DDH rat model (Supplementary Fig. 1a). Body weight of the DA-DDH rats was approximately 10% lower than that of the control rats (Supplementary Fig. 1b). The radiograms revealed hip dislocation and the hypoplastic acetabulum in the DA-DDH rats (Fig. 1a). Indeed, macroscopic assessment of the 4-week-old DA-DDH rats showed that the acetabulum cavity was shallow and the femoral head was small and oval shaped, compared with those in the control rats (Fig. 1b). Histologically, in the 10-day-old DA-DDH rats, the femoral head was dislocated; however, safranin O (SO) staining in the acetabular surface was not decreased, compared with that in the control rats (Supplementary Fig. 2a). In the 4-week-old DA-DDH rats, the acetabular surface and femoral head were not in contact, the joint cavity was filled with fibrous tissue, and SO staining in the acetabular surface was less intense than that in the control rats (Fig. 1c). In both 10-day- and 4-week-old DA-DDH rats, the anterior wall of the acetabulum had atrophied.

The expression of type II collagen α1 (*Col2a1*), aggrecan (*Acan*), type X collagen α1 (*Col10a1*), matrix metalloproteinase 13 (*Mmp13*), and type I collagen α1 (*Col1a1*) in the acetabular cartilage of the 10-day-old DA-DDH rats was comparable to that in the control rats (Supplementary Fig. 2b). The expression of *Col2a1*, *Acan,* and *Col10a1* was downregulated, but the expression of *Mmp13* and *Col1a1* did not significantly change in the 4-week-old DA-DDH rats compared to that in the control rats (Fig. 1d). In situ hybridization analyses showed no differences in *Col2a1* and *Col1a1* expression between the 10-day-old control and DA-DDH rats (Supplementary Fig. 2c) but revealed downregulated expression of *Col2a1* and upregulated expression of *Col1a1* in the acetabular cartilage of the 4-week-old DA-DDH rats (Fig. 1e). Second-harmonic generation (SHG) signal of the acetabular superficial cartilage was weaker in the 10-day-old DA-DDH rat than in the control rat (Supplementary Fig. 2d). The terminal deoxynucleotidyl transferase-mediated dUTP nick end labeling (TUNEL) assay and immunostaining for Ki67 showed that apoptosis and cell proliferation in the acetabular cartilage of the 10-day and 4-week-old DA-DDH rats were comparable to those in the control rats (Supplementary Fig. 2e,f).

### Total RNA-sequencing (RNA-seq) analysis of the acetabular cartilage of the DA-DDH rats

**Figure 2:**
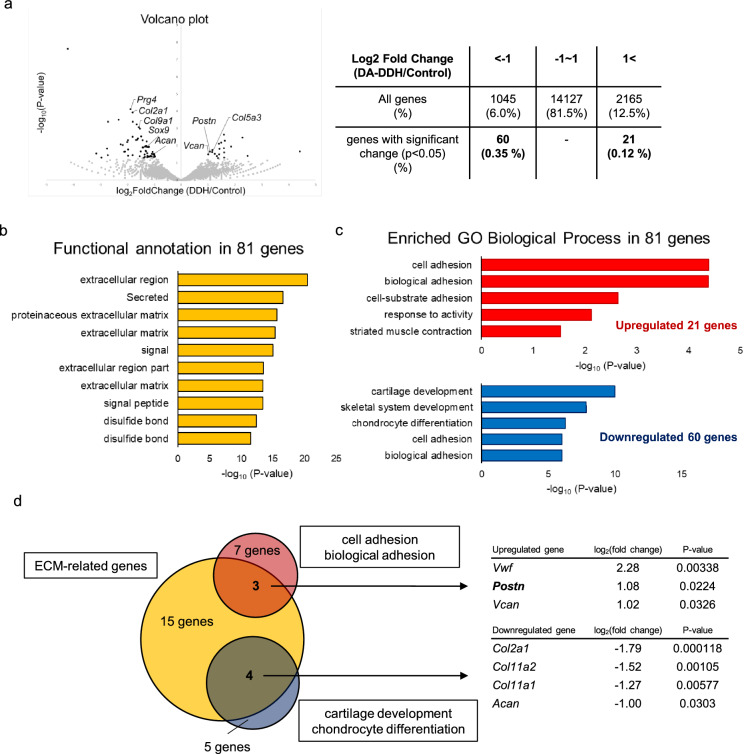
RNA-sequencing analysis of the acetabular cartilage of 3-week-old DA-DDH rats. (**a**) Volcano plot for RNA-sequencing data obtained from the acetabular cartilage of 3-week-old rats. Control and DA-DDH groups involved bilateral acetabulum pooled from 4 and 5 rats, respectively. Black dots indicate significantly (P < 0.05) and differentially expressed genes in the DA-DDH rats whose expression was modulated by more than twofold. The table summarizes the number and percentage of differentially expressed (upper row) and significantly upregulated and downregulated expressed genes (lower row). (**b**) Gene Ontology (GO) analyses were performed using the DAVID bioinformatics resource. Functional annotation clustering of keywords among the GO terms for 81 genes that were significantly differentially expressed between the control and DA-DDH groups (P < 0.05). (**c**) The enriched GO Biological Process in 21 significantly upregulated and 60 significantly downregulated genes in DA-DDH rats is illustrated by − log_10_(P-value). (**d**) Genes related to cell adhesion and chondrogenesis were detected among 15 extracellular region/ECM-related genes in DA-DDH rats. The table shows the expression of cell adhesion and chondrogenic marker genes in the extracellular region/ECM-related genes.

Of genes whose expression levels differed between the 3-week-old control and DA-DDH rats by more than twofold, 81 genes were significantly differentially expressed between the two groups (P < 0.05). Among these, the expression levels of 21 genes were upregulated, where those of 60 genes were downregulated. The mechano-sensitive gene, *Prg4*, was among the 60 genes with downregulated expression (Fig. 2a). Gene Ontology (GO) analyses of these 81 genes showed functional annotation clustering of keywords such as “extracellular region,” “proteinaceous extracellular matrix (ECM),” “ECM,” and “extracellular region part” (Fig. 2b). Cell adhesion-related and biological adhesion-related genes were enriched among the 21 genes whose expression levels were upregulated in the DA-DDH rats. In contrast, cartilage development and chondrocyte differentiation-related genes were enriched among the 60 genes whose expression levels were downregulated in the DA-DDH rats (Fig. 2c). Three cell adhesion-related genes with upregulated expression and four chondrogenesis-related genes with downregulated expression were detected among the ECM-related genes in DA-DDH rats (Fig. 2d).

### Expression of Postn and catabolic factors in acetabular cartilage of DA-DDH rats

**Figure 3:**
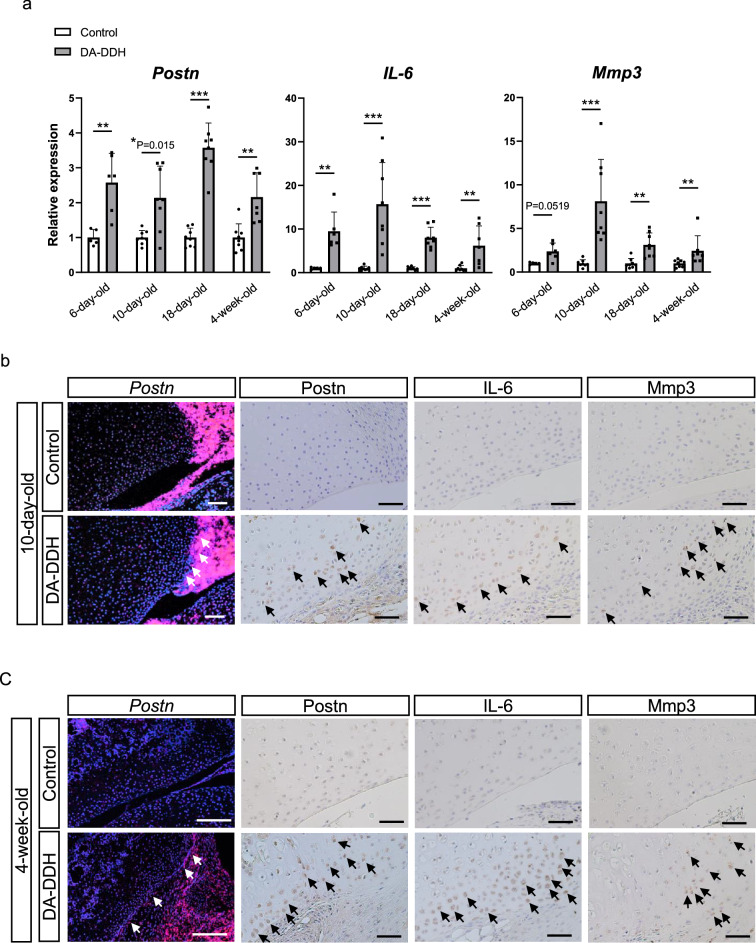
Periostin (Postn), interleukin-6 (*IL-6*), and matrix metalloproteinase 3 (*Mmp3*) expression in the acetabular cartilage of the control and DA-DDH rats. (**a**) Relative mRNA expression of *Postn*, *IL-6,* and *Mmp3* in the acetabular cartilage of 6-day- (n = 5; control, n = 6; DA-DDH), 10-day- (n = 8; control, n = 8; DA-DDH), 18-day- (n = 8; control, n = 8; DA-DDH) and 4-week-old rats (n = 8; control, n = 7; DA-DDH). (**b**) In situ hybridization and immunostaining of the acetabulum of 10-day-week-old rats. (**c**) In situ hybridization and immunostaining of the acetabulum of 4-week-old rats. The white and black arrows mark *Postn* expression and Postn, IL-6, and Mmp3 positive cells, respectively. Scale bar: 10-day-old; 100 μm, 4-week-old; 200 μm. The Mann–Whitney *U* test was used for statistical analysis. Values indicate the mean ± SD. *P < 0.05, **P < 0.01, ***P < 0.001.

*Postn*, interleukin 6 (*IL-6)*, and matrix metalloproteinase 3 *(Mmp3)* expression levels were upregulated in the acetabular cartilage of the 6-day-, 10-day-, 18-day- and 4-week-old DA-DDH rats (Fig. 3a). *IL-1β* and *TNFα* expression was not upregulated in the DA-DDH rats. *Nos2* expression was upregulated in the 4-week-old DA-DDH rats (Supplementary Fig. 3a). Immunostaining revealed upregulation of Postn, IL-6, and Mmp3 expression in the acetabular cartilage and fibrous tissues in contact with the cartilage of the 10-day- and 4-week-old DA-DDH rats (Fig. 3b,c and Supplementary Fig. 3b).

### Role of Postn in articular chondrocytes

**Figure 4:**
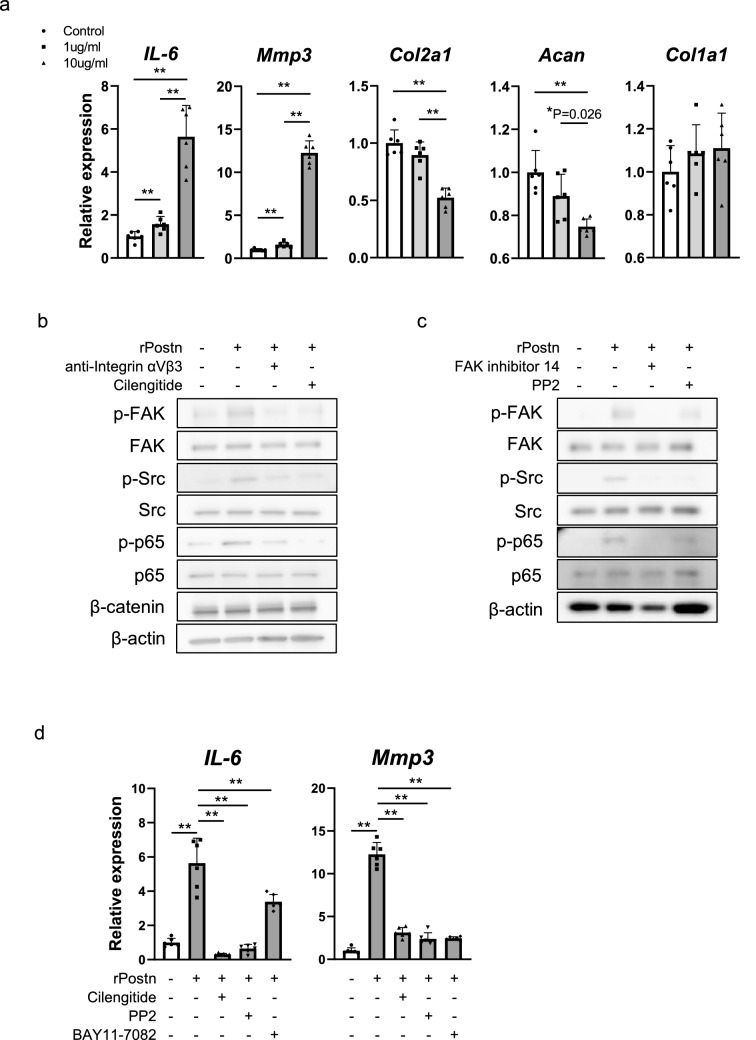
Postn suppressed *Col2a1* and *Acan* expression and promoted *IL-6* and *Mmp3* expression by activating the integrin-focal adhesion kinase (FAK)-Src-nuclear factor κB (NF-κB) signaling pathway in chondrocytes. (**a**) Relative mRNA expression of *IL-6*, *Mmp3*, *Col2a1*, *Acan* and *Col1a1* in chondrocytes treated with recombinant Postn (rPostn) (0, 1, and 10 μg/ml) for 24 h (n = 6 per group). (**b**) Western blot analysis for evaluating p-FAK, FAK, p-Src, Src, p-p65, p65, and β-catenin expression in rat primary chondrocytes treated with rPostn (1 μg/ml) alone or together with integrin αVβ3 antibody or Cilengitide (2.5 μM) for 1 h. (**c**) Western blot analysis to detect the expression of p-FAK, FAK, p-Src, Src, p-p65, and p65 in rat primary chondrocytes treated with rPostn (1 μg/ml) alone or together with FAK inhibitor 14 (2.5 μM) or PP2 (2.5 μM) for 1 h. (**d**) Relative mRNA expression of *IL-6* and *Mmp3* in rat primary chondrocytes treated with rPostn (10 μg/ml) alone or together with Cilengitide, PP2, or BAY11-7082 (5 μM) for 24 h (n = 6 per group). The Kruskal–Wallis test was used for multiple comparisons. The Mann–Whitney *U* test was used for comparisons between two groups. Values indicate the mean ± SD. *P < 0.05, **P < 0.01, ***P < 0.001.

We confirmed that *IL-6* and *Mmp3* expression was upregulated, and *Col2a1* and *Acan* expression was downregulated in rat primary chondrocytes treated with rPostn (Fig. 4a). We showed that the phosphorylated focal adhesion kinase (p-FAK), phosphorylated Src (p-Src), and phosphorylated p65 (p-p65) level was increased in rPostn-treated chondrocytes. In contrast, the expression of β-catenin remained unchanged. The increased levels of p-FAK, p-Src, and p-p65 induced by rPostn were suppressed by integrin αVβ3 antibody and cilengitide, an integrin αVβ3 and αVβ5 inhibitor (Fig. 4b). The increased levels of p-p65 induced by rPostn were suppressed by FAK inhibitor 14 and PP2, an inhibitor of Src (Fig. 4c). Furthermore, the expression of *IL-6* and *Mmp3* upregulated by rPostn was suppressed by cilengitide, PP2, and BAY11-7082, an inhibitor of NF-κB signaling (Fig. 4d).

### *Postn* expression induced by dynamic load reduction is mediated via STAT3 signaling

**Figure 5:**
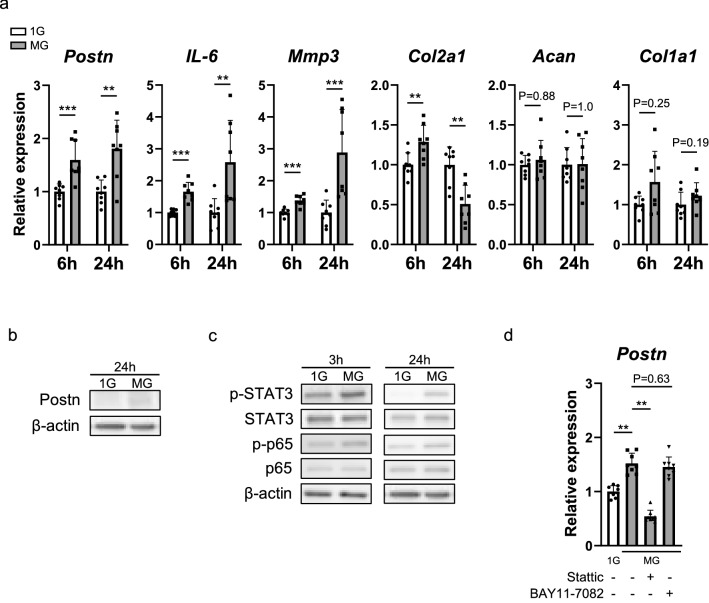
Microgravity (MG) induced Postn expression through signal transducer and activator of transcription 3 (STAT3) phosphorylation. (**a**) Relative mRNA expression of *Postn*, *IL-6*, *Mmp3*, *Col2a1*, *Acan*, and *Col1a1* in chondrocytes cultured for 6 and 24 h under 1 g (1G) centrifugal force (1G) and in MG. (n = 8 per group). (**b**) Western blot to detect Postn expression in rat primary chondrocytes cultured for 24 h in 1G and MG. (**c**) Western blot analysis for evaluating p-STAT3, STAT3, p-p65, and p65 expression in rat primary chondrocytes cultured for 3 and 24 h in 1G and MG. β-actin was used as an internal control. (**d**) Real-time qPCR results for *Postn* in rat primary chondrocytes cultured in 1G and MG in the presence or absence of Stattic (2.5 μM) or BAY11-7082 for 6 h. (n = 8 per group). The Mann–Whitney *U* test (**a**) and Kruskal–Wallis test (**d**) were used for statistical analysis. Values indicate the mean ± SD. *P < 0.05, **P < 0.01, ***P < 0.001.

We cultured rat primary chondrocytes under a virtual microgravity (MG) state using an RPM (Supplementary Fig. 4a). The expression levels of Postn in the chondrocytes cultured under MG were upregulated, compared with that in the chondrocytes cultured under 1G condition (Fig. 5a,b). The expression levels of *IL-6* and *Mmp3* were upregulated, that of *Col2a1* was downregulated, and those of *Acan* and *Col1a1* remained unchanged in the chondrocytes cultured in MG (Fig. 5a). The expression of phosphorylated STAT3 (p-STAT3) was higher in chondrocytes cultured in MG for 3 h than in chondrocytes cultured under 1G condition, but the expression of p-p65, p-p38, p-Erk1/2, and β-catenin remained unchanged (Fig. 5c and Supplementary Fig. 4b). The expression of p-STAT3 and p-p65 was higher in chondrocytes cultured in MG for 24 h than under 1G condition (Fig. 5c). Furthermore, the increased expression of *Postn* in chondrocytes cultured under MG was suppressed in chondrocytes treated with Stattic, an inhibitor of STAT3 signaling. However, a similar suppression was not observed in cells treated with BAY11-7082 (Fig. 5d).

### *IL-6* induces *Postn* expression via STAT3 signaling by a feedback mechanism

**Figure 6:**
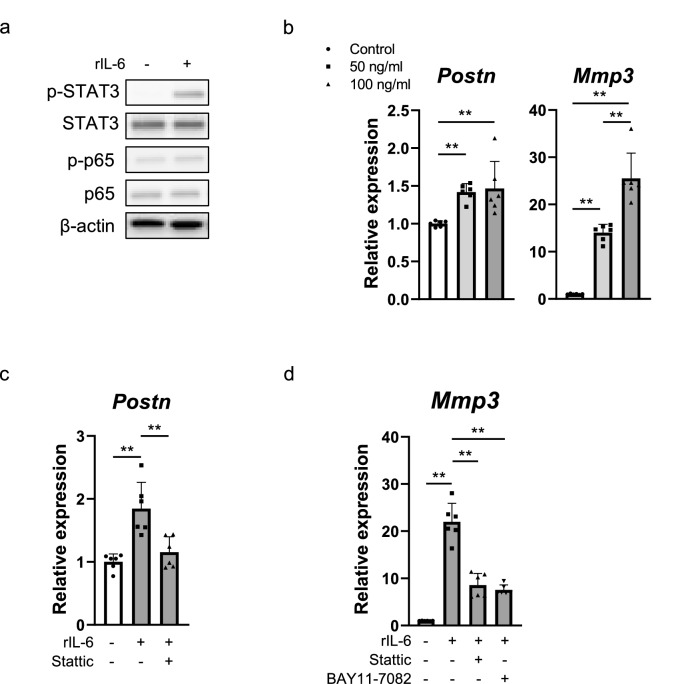
IL-6 accelerated STAT3-Postn-NF-κB signaling. (**a**) Western blot results for p-STAT3, STAT3, p-p65, and p65 expression in rat primary chondrocytes treated with recombinant IL-6 (rIL6; 50 ng/ml) for 2 h. (**b**) Real-time qPCR results for *Postn* and *Mmp3* expression in rat primary chondrocytes treated with rIL-6 (0, 50, 100 ng/ml) for 24 h (n = 6 per group). (**c**) Real-time qPCR results for *Postn* expression in rat primary chondrocytes treated with rIL-6 (50 ng/ml) alone or together with Stattic for 24 h (n = 6 per group). (**d**) Real-time qPCR results for *Mmp3* expression in rat primary chondrocytes treated with rIL-6 (50 ng/ml) alone or together with Stattic or BAY11-7082 for 24 h (n = 6 per group). The Kruskal–Wallis test was used for multiple comparisons. The Mann–Whitney *U* test was used for comparisons between two groups. Values indicate the mean ± SD. *P < 0.05, **P < 0.01, ***P < 0.001.

The expression levels of p-STAT3 were upregulated in rat primary chondrocytes treated with rIL-6 (Fig. 6a). Additionally, the expression of *Postn* and *Mmp3* was upregulated in chondrocytes treated with rIL-6 in a dose-dependent manner (Fig. 6b). Furthermore, rIL-6-induced upregulated expression of *Postn* was suppressed by Stattic (Fig. 6c), and rIL-6-induced upregulated expression of *Mmp3* was suppressed by Stattic and BAY11-7082 (Fig. 6d). Moreover, rIL-6-induced upregulated expression of Mmp3 was suppressed in *Postn*^*−/−*^ mouse-derived primary chondrocytes when compared with WT mouse-derived primary chondrocytes (Supplementary Fig. 5).

### Cartilage degeneration is suppressed in *Postn*^−/−^ DA-DDH mice

**Figure 7:**
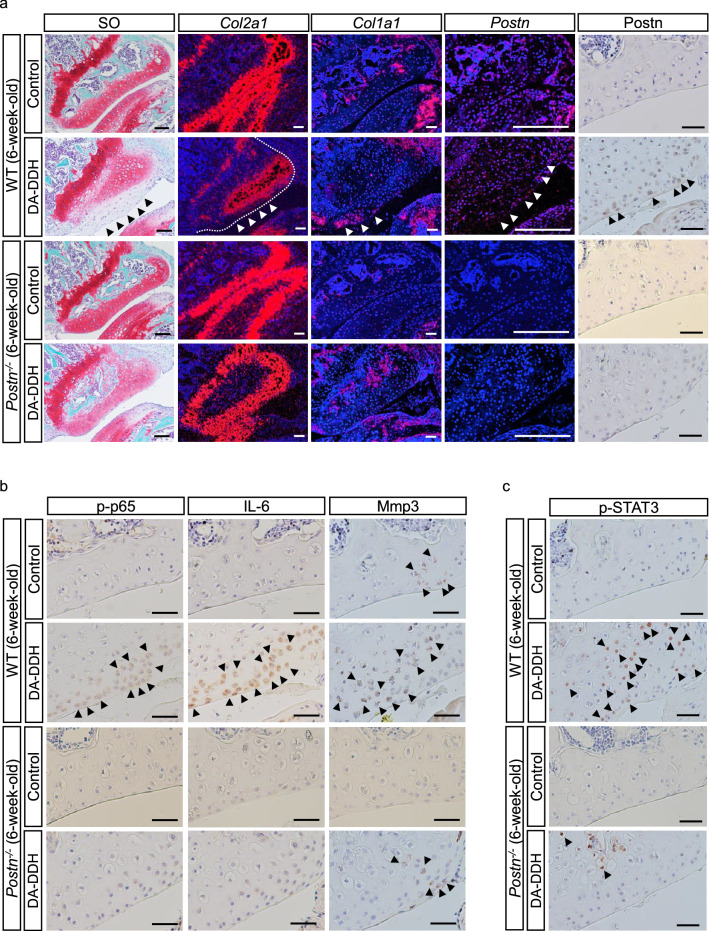
Postn deficiency suppressed cartilage degeneration in the acetabulum of DA-DDH mice. (**a**) SO staining, in situ hybridization (*Col2a1*, *Col1a1*, and *Postn*), and immunostaining (Postn) of the anterior wall of the acetabulum in 6-week-old mice. The white dotted line in the *Col2a1 *in situ hybridization indicates the joint line of the acetabulum. The black and white arrowheads indicate a fibrous change of cartilage, downregulated expression of *Col2a1*, upregulated expression of *Col1a1,* and Postn positive cells. Scale bar: SO staining; 100 μm, in situ hybridization; 200 μm, immunostaining; 50 μm. (**b**) Immunostaining for p-p65, IL-6, and Mmp3 expression of the anterior wall of the acetabulum in the 6-week-old mice. The black arrowheads indicate p-p65-, IL-6-, and Mmp3-positive cells. Scale bar: 50 μm. (**c**) Immunostaining for p-STAT3 expression in the anterior wall of the acetabulum in 6-week-old mice. Scale bar: 50 μm.

**Figure 8:**
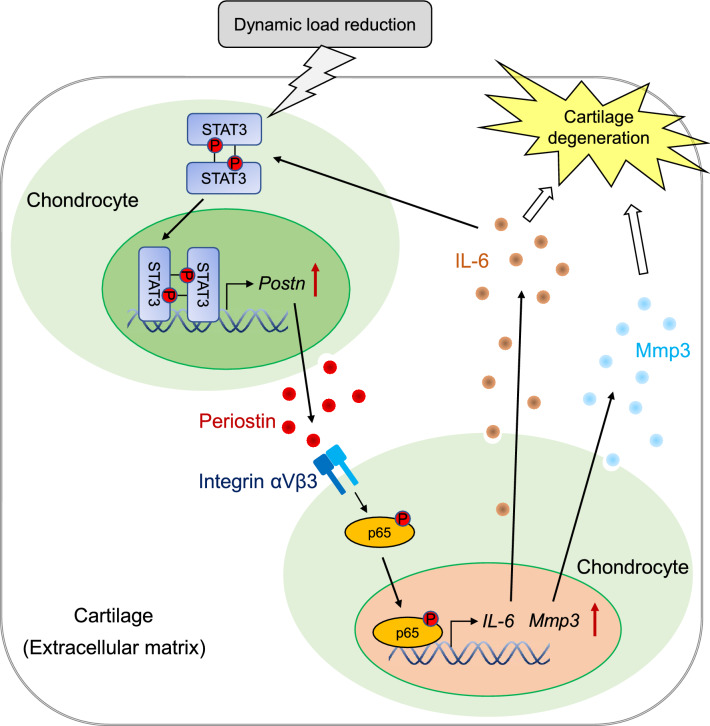
Schematic illustration of acetabular cartilage degeneration by IL-6 and MMP3 via the STAT3/periostin/NF-κB axis in developmental dysplasia of the hip. Dynamic load reduction induces Postn expression by activating STAT3 signaling; Postn induces IL-6 and Mmp3 expression through integrin-NF-κB signaling in the articular cartilage. IL-6/STAT3 signaling induced by Postn accelerates a feedback mechanism.

We generated the DA-DDH mouse model (Supplementary Fig. 6a). Although the hip joint of 8-week-old DA-DDH mice was not completely dislocated, the acetabulum and the femoral head had poor compatibility, the anterior and inferior walls of acetabulum were hypoplasia, the posterior wall was thickened, and the femoral head was flattening (Supplementary Fig. 6b,c). These phenotypes were similar with the rat DA-DDH model. In *Postn*^*−/−*^ DA-DDH mice, cartilage degeneration in the anterior wall of the acetabulum was markedly suppressed relative to that in WT DA-DDH mice (Fig. 7a and Supplementary Fig. 7a). Additionally, the expression of Col2a1 was downregulated, whereas the expression of Col1a1 and Postn was upregulated, in the acetabular cartilage of the WT DA-DDH mice (Fig. 7a and Supplementary Fig. 7b). The downregulation of Col2a1 expression and upregulation of Col1a1 expression were suppressed in the acetabular cartilage of the *Postn*^−/−^ DA-DDH mice. Furthermore, the expression of Postn, p-p65, IL-6, and Mmp3 was upregulated in the acetabular cartilage of the WT DA-DDH mice, but this upregulated expression was suppressed in the *Postn*^−/−^ DA-DDH mice (Fig. 7a,b and Supplementary Fig. 7c). Immunostaining revealed that p-STAT3 expression was increased in the acetabular cartilage of the WT DA-DDH mice but suppressed in the *Postn*^−/−^ DA-DDH mice (Fig. 7c and Supplementary Fig. 7d), indicating that downstream targets of Postn could stimulate STAT3 signaling. These results showed that the loss of Postn expression protected against cartilage degeneration in the acetabulum of DA-DDH models by suppressing the expression of IL-6 and Mmp3 via NF-κB signaling. Thus, we demonstrated that dynamic load reduction in the dislocated hip associated with DDH induced acetabular cartilage degeneration by activating the STAT3/Postn/NF-κB/IL-6 and Mmp3 feedback loop (Fig. 8).

## Discussion

This study focused on the mechanism by which cartilage degeneration is induced in articular cartilage exposed to reduced dynamic load. *Prg4* expression was downregulated in the acetabular cartilage of DA-DDH rats. The anterior wall of the acetabulum in DA-DDH models was atrophied. SHG can evaluate collagen orientation in unstained tissue sections^20^. Moreover, it can identify changes in collagen orientation before changes in gene expression in tissues take place. Although SHG signaling intensity in cartilage subjected to excessive stress or traumatic damage is stronger than that under normal conditions^20^, in our study, the SHG signal of the cartilage of the anterior wall of the acetabulum in the DA-DDH rat was weaker than that in the control rat. Furthermore, wear, fibrillation and lack of hyaline cartilage observed in traumatic knee OA models such as destabilization of the medial meniscus model were not observed in the acetabular cartilage of DA-DDH models, but a fibrous change of hyaline cartilage was observed in their acetabular cartilage. Based on these results, we inferred that dynamic load on the acetabular cartilage in the dislocated hip of DA-DDH rats, especially on the cartilage of the atrophied anterior wall, was reduced. Therefore, we suggest that mechanisms different from excessive stress or traumatic damage-induced OA were involved in cartilage degeneration in the dislocated hip of DDH.

Postn is expressed in tissue exposed to load, as well as in damaged tissue, and promotes cell proliferation and migration, dedifferentiation, and tissue degeneration and reconstruction by activating several signaling pathways^13,16,17,21,22^. Postn interacts with other ECM molecules and induces fibrous tissue growth to contribute to the maintenance of tissue structure^13^. Although the Postn expression level is low in normal cartilage^23^, its expression is upregulated in damaged cartilage of patients with knee OA and rodent models of traumatic OA^16,17^. Han et al. indicated that Postn promoted cartilage degeneration via DDR1-Akt/β-catenin signaling^24^. Attur et al*.* demonstrated that Postn-induced MMP13 expression in chondrocytes was suppressed by inhibiting Wnt/β-catenin signaling and that loss of Postn protected against cartilage degeneration in models of traumatic knee OA^14,16^. In addition, Postn expression induced by damaged anterior cruciate ligament reportedly causes cartilage degeneration via paracrine effects^25^. Fibroblast-derived Postn promotes colorectal tumorigenesis via integrin-FAK-Src signaling pathway; then, IL-6 induced by Postn accelerates Postn expression by stimulating STAT3 signaling^26^. Bone marrow mesenchymal stromal cell-derived Postn promotes acute lymphoblastic leukemia progression by activating the integrin/ILK/NF-κB/CCL2/STAT3 loop^27^. Thus, Postn is involved in cell-to-cell interaction and regulates several intracellular signaling pathways.

We also revealed that the expression of Postn, IL-6, and Mmp3 was upregulated and that of *Col2a1* and *Acan* was downregulated in the articular cartilage of the unloaded acetabulum in DA-DDH models, compared with that in controls. In addition, Postn induced the expression of *IL-6* and *Mmp3* via integrin-FAK-Src-NF-κB signaling. We also showed that Postn suppressed the expression of *Col2a1* and *Acan* in chondrocytes. *IL-6* is a pro-inflammatory cytokine and induces the degeneration of various ECM components^28–31^. *MMP3* is a type of stromelysin that plays an essential role in tissue remodeling by degenerating ECM^32^ and inducing the breakdown of proteoglycans in the cartilage in OA and rheumatoid arthritis^33,34^. The expression of *MMP3* is reportedly upregulated in the articular cartilage of DDH-related hip OA patients^35^. We showed that the expression levels of IL-6 and Mmp3 were downregulated and cartilage degeneration was suppressed in the acetabular articular cartilage of the *Postn*^−/−^ DA-DDH mice compared with those of the WT DA-DDH mice.

To reproduce in vivo conditions in an in vitro culture system such that the chondrocytes were not stimulated with sufficient mechanical load, we cultured chondrocytes under a virtual MG state using an RPM. We observed that dynamic load reduction induced Postn expression via STAT3 signaling. In addition, the expression of *Col2a1* was downregulated in chondrocytes cultured under the MG condition. Moreover, IL-6, a downstream target of Postn, upregulated Postn expression via STAT3 signaling. Our findings provide supporting evidence that reduced dynamic load can promote acetabular cartilage degeneration by activating the STAT3/Postn/NF-κB/IL-6 and Mmp3 feedback loop in an autocrine or paracrine manner and may suppress chondrogenesis during the hip dislocation of DDH.

This study has a few limitations. We did not consider genetic^36–40^ or embryonic factors. DA-DDH rats showed approximately 10% lower body weight than the control rats. In DA-DDH models, nutritional deficiency, inflammation, tissue contusion, ischemia, wear and tears around the joint caused by swaddling may have affected cartilage homeostasis. Additionally, loss of synovial fluid might be biologically harmful to acetabular chondrocytes due to the loss of nutrition and synovial fluid derived stem cells, which are important to cartilage repair. The impaired perichondrium, a chondrocyte progenitor cell niche, might also be involved. Furthermore, our in vivo results suggest that Postn expression in the acetabular cartilage might be induced by the paracrine effect of Postn derived from fibrous tissue in and around the hip joint in DA-DDH models. Therefore, in future studies, Postn expression should be disrupted in a temporally controlled and cartilage-specific manner by genetic ablation in postnatal mice to investigate the acetabular cartilage after hip dislocation. Acetabular hypoplasia and hip dislocation occur to varying degrees during the natural process of DDH. However, we did not examine the degree and incidence of hip dislocation and acetabular dysplasia of rats with swaddling in detail. The mechanism of cartilage degeneration in mild hip dysplasia may differ from that of complete hip dislocation. In DA-DDH mice, although the hip joint did not completely dislocate, subluxation between the acetabulum and the femoral head caused similar changes with DA-DDH rats. *Postn*^−/−^ mice exhibit some skeletal abnormalities, which complicate mechanistic exploration in cartilage^14^. In the future, acetabular cartilage with DDH of various degrees should be investigated and compared to non-DDH to validate the underlying mechanism.

In conclusion, we demonstrated that dynamic load reduction in the dislocated hip associated with DDH induced acetabular cartilage degeneration by activating the STAT3/Postn/NF-κB/IL-6 and Mmp3 feedback loop in an autocrine or paracrine manner. We showed one pathway of mechanical–biological processes of chondrocytes in cartilage degeneration associated with DDH, but further studies are needed for understanding complex biological processes involving many cell types in DDH.

## Methods

### Animals

All methods were performed in accordance with the relevant guidelines and regulations. All experimental protocols were approved by the Gifu University Animal Experiment Committee (H30-099). The animal experiments were conducted in compliance with the Animal Research: Reporting in vivo experimental guidelines. Information on the animals used has been provided in Supplementary Table 1. *Postn*^−/−^ mice were provided by the Material Management Center in Kyushu University^13^. We crossed these mice and C57BL/6J mice to generate the *Postn*^−/−^ and *Postn*^+*/*+^ littermate controls. Rats and mice were housed in individually ventilated cages in a hygienic barrier facility operating at 23 ± 1 °C and 50 ± 10% humidity. The controls and DA-DDH models used for in vivo analyses were littermate and housed in the same cages. Food and water were available freely, and photoperiod lasted from 8:00 to 20:00.

### Establishment of animal DA-DDH models

The DA-DDH rats were generated as described previously^11,12,41,42^. Since the lower extremities of the neonate mice were too short for swaddling, to establish the DA-DDH mice, we threaded 5–0 prolene (Ethicon) onto the distal femur and proximal tibia of two-week-old C57BL/6 mice, tied them, and swaddled in hip adduction and knee extension positions for two weeks. The surgical tape for swaddling was changed in the DA-DDH models once every two days, and they were released from the fixing for several hours at that time. Water bottles and food were placed on the floor of the cages. More than three rats or mice were used in each group to obtain unbiased and reliable results; the sample size for each experiment is indicated in the figures representing the corresponding results. Rats and mice were divided into DA-DDH and control groups by randomization; sex was not evaluated. The body weight of the rats was evaluated over time. A few rats and mice had to be excluded from the evaluation owing to mortality due to gastrointestinal disorders induced by abdominal compression, but no other exclusion criteria were set. Flow charts for the rat and mouse in vivo experiments were showed in Supplementary Figs. 1a and 5a. The numbers of animals used in this study are summarized in Supplementary Table 2.

### X-ray examination

Radiographic examination was performed with a Faxitron cabinet X-ray system (Model 43855D, Faxitron X-Ray LLC), at 26 kV and exposure time of 10 s.

### Histological analyses

The hip joints were fixed in 4% paraformaldehyde in phosphate-buffered saline (PBS, pH 7.4) overnight at 4 °C and decalcified with G-Chelate Mild (pH 7.2 EDTA buffer; GenoStuff) for 14 days. The hip joints were fixed in paraffin, sectioned into 5-μm-thick axial sections, and stained with Hematoxylin–eosin and safranin O/fast green.

### Acetabular cartilage collection and RNA extraction

Acetabular cartilage samples were collected using tools for microsurgery and a microscope to avoid collecting subchondral bone, bone marrow, and soft tissues as possible. Total RNA was extracted using the RNeasy Plus Mini Kit (Qiagen) according to the manufacturer’s instructions. Independent RNA samples were extracted from the bilateral acetabulum of each model.

### RNA-seq analysis

Total RNA-seq was performed as described previously^43–45^. The integrity of the isolated RNA was verified using the 2100 Bioanalyzer (Agilent Technologies). RNA samples with an RNA integrity value > 8 were diluted to 100 ng/ml before further analyses. RNA-Seq libraries were prepared using the TruSeq Stranded mRNA Sample Prep Kit set A (Illumina). Each library was sequenced on MiSeq (Illumina) with MiSeq Reagent kit V3 150 cycle (Illumina) by 75 base pair-end reads. Differentially expressed genes were determined by an exact test after normalization. Pathway analyses were performed using DAVID Bioinformatics Resources as described previously^46^.

### Real-time quantitative PCR (qPCR)

The extracted RNA was reverse-transcribed using the High-Capacity cDNA Transcription Kit (Applied biosystems). Real-time qPCR reactions were prepared using TB Green Premix Ex Taq II (Takara Bio) and run on the Thermal Cycler Dice Real-Time System II (Takara Bio). The reactions were performed in triplicate, and target mRNA levels were normalized with those of glyceraldehyde-3-phosphate dehydrogenase used as the internal control. PCR primers are listed in Supplementary Table 3.

### In situ hybridization

In situ hybridization using ^35^S labeling of an RNA-probe was performed as described previously^47^. The hybridization signals were imaged using a red filter, and the images were then superimposed on blue fluorescent images of the cell nuclei stained with Hoechst 33258 dye (Sigma-Aldrich). Additional information on the reagents used has been provided in Supplementary Table 1.

### SHG analysis

SHG imaging was performed by multi-photon microscopy (A1R-MP, Nikon, Inc.) wherein the microscope was equipped with a water immersion lens (CFI75 Apo 25 × W MP, NA:1.1, Nikon, Inc.) and a Ti:sapphire laser oscillator (MaiTai eHP, Spectra-Physics, Inc.) as described previously^20,48^. Excitation wavelengths of 950 nm with emission filter sets, the dichroic mirror 495 nm, and the shortpass filter 492 nm were used to detect the SHG signal. The images were acquired as z-stack image sequences with a step size of 2 μm. For observing whole tissue sections, 4 × 4 or 5 × 5 images (each 0.5 mm × 0.5 mm field of view, size 512 × 512 pixels) were recorded and stitched to create large images using NIS Elements software (Nikon, Inc.). The images were originally recorded as 12-bit gray-level images and subjected to the maximum intensity projection.

### Immunohistochemistry analysis

The sections were deparaffinized, rehydrated, and then treated with Liberate Antibody Binding Solution (Thermo Fisher Scientific) for 10 min and blocked with 3% bovine serum albumin (BSA) in PBS. The sections were then incubated overnight at 4 °C with the primary antibodies. The signal was detected using the En Vision Detection Kit (Dako). For assessing Ki67 expression using immunofluorescence, deparaffinized sections were boiled for 10 min under high pressure and temperature in Histofine (Nichirei Bioscience) was used for antigen retrieval. The antibodies used in these experiments are listed in Supplementary Table 1.

### TUNEL assay

The TUNEL assay was performed using the In Situ Cell Death Detection Kit, AP (Sigma-Aldrich), according to the manufacturer’s instructions.

### Primary culture of chondrocytes

Chondrocytes were isolated from 5–7-day-old rats according to a protocol described previously^49^. The cells were cultured in high-glucose Dulbecco’s Modified Eagle’s Medium supplemented with 10% fetal bovine serum (Thermo Fisher Scientific) and 1% penicillin–streptomycin (FUJIFILM Wako).

### Chondrocytes treated with recombinant protein and inhibitors

Primary chondrocytes were seeded on 24-well culture plates (6 × 10^4^ cells/well) for RNA extraction or on 6-well culture plates (3 × 10^5^ cells/well) for protein extraction and incubated overnight. The cells were incubated in a fresh serum-free medium with recombinant proteins and/or inhibitors. The reagents used in this experiment are listed in Supplementary Table 1. For the control group, the cells were treated with the same volume of the vehicle.

### Three-dimensional microgravity cell culture and two-dimensional fast-rotating culture

The MG condition was generated using an RPM device (Yamato Scientific). The chondrocytes were seeded (3.5 × 10^5^ cells/flask) on T25 flasks (Corning) and cultured overnight. The flasks were filled with culture medium without air bubbles and fixed on the RPM, positioned in an incubator set at 37 °C and supplied with 5% CO_2_. A centrifugal force of 1 g (1G) was established for the control.

### Western blotting

Protein was extracted from cells using RIPA buffer supplemented with a Halt Protease and Phosphatase Inhibitor Cocktail (Thermo Fisher Scientific) and centrifuged; the supernatant was collected. An equal amount of lysate (20 μL), containing 5 μg protein, was loaded and resolved on a 10% SDS polyacrylamide gel (FUJIFILM Wako). The proteins were transferred onto a nitrocellulose membrane (Merck Millipore). The membranes were blocked using 3% BSA in Tris-buffered saline with Tween 20 and incubated overnight with the primary antibody. Antibodies used in this experiment are listed in Supplementary Table 1. The membranes were then incubated with a secondary HRP-linked antibody and incubated with ImmunoStar Zeta (FUJIFILM Wako) for generating the immunoreactive signal. The bands were detected with ImageQuant™ LAS 4000 (GE Healthcare).

### Statistical analysis

All results are presented as means ± SD. The sample size was determined based on previous studies^27^ and was not calculated based on statistical power. Because of the small sample size, we could not guarantee the normality and homogeneous variance of each variable. Therefore, a non-parametric method was used for all statistical analyses. All results are presented as the median and interquartile range (IQR). The data were evaluated using a Mann–Whitney *U* test and Kruskal–Wallis test using GraphPad PRISM software version 8.0 (GraphPad, Inc.). The statistical test used in each experiment is indicated in the corresponding figure. A two-sided *P*-value less than 0.05 was considered to reflect statistically significant differences.

## Supplementary Information

Supplementary Information.

## Data Availability

The RNA-seq data have been deposited in the Gene Expression Omnibus database under the accession code GSE173637. All other data supporting the findings of this study are available within the article, its Supplementary information files, and from the corresponding author upon reasonable request.
