# Supplementary material for: Reduced dynamic loads due to hip dislocation induce acetabular cartilage degeneration by IL-6 and MMP3 via the STAT3/periostin/NF-κB axis
Source: Sci Rep. 2022 Jul 16;12:12207. doi: 10.1038/s41598-022-16585-6 (PMC9288549; doi:10.1038/s41598-022-16585-6)
Supplement: Supplementary file 1 — Supplementary Information. [file 41598_2022_16585_MOESM1_ESM.pdf]

Reduced dynamic loads due to hip dislocation induces acetabular cartilage degeneration by IL-6 and MMP3 via STAT3/periostin/NF-κB axis

Yutaka Nakamura,[1] Mitsuru Saitou,[1] Shingo Komura,[1] Kazu Matsumoto,[1] Hiroyasu Ogawa,[1] Takaki Miyagawa,[1] Takashi Saitou,[2] Takeshi Imamura,[2] Yuuki Imai,[3] Hiroshi Takayanagi,[4] and Haruhiko Akiyama[1]

[1] Yutaka Nakamura, MD, Mitsuru Saitou, MD, Shingo Komura, MD, PhD, Kazu Matsumoto, MD, PhD, Hiroyasu Ogawa, MD, PhD, Takaki Miyagawa, MD, PhD, Haruhiko Akiyama, MD, PhD: Department of Orthopaedic Surgery, Graduate School of Medicine, Gifu University, Gifu, Japan

[2] Takashi Saitou, PhD, Takeshi Imamura, MD, PhD: Department of Molecular Medicine for Pathogenesis, Graduate School of Medicine, Ehime University, Ehime, Japan.

[3] Yuuki Imai, MD, PhD: Division of Integrative Pathophysiology, Proteo-Science Center, Ehime University, Ehime, Japan.

[4] Hiroshi Takayanagi, MD, PhD: Department of Immunology, Graduate School of Medicine and Faculty of Medicine, The University of Tokyo, Tokyo, Japan

Address correspondence to Haruhiko Akiyama, MD, PhD, Department of Orthopaedic Surgery, Graduate School of Medicine, Gifu University, Gifu 501-1194, Japan
E-mail: hakiyama@gifu-u.ac.jp

Conflict of interest
The authors have no conflict of interest to declare.

Supplementary Information

Legends to Supplementary Figures (Supplementary Fig. 1 – 13)

Legends to Supplementary Tables (Supplementary Table 1 – 3)

Supplementary Fig. 1.

Establishment of rat disarticulation models of developmental dysplasia of the hip (DA-DDH).

(a) Flow chart for the rat *in vivo* experiments. Neonatal rats were randomly devided into two groups. Rats in DA-DDH group were swaddled for 10 days.

(b) Body weight of the control and DA-DDH rats over time.

Supplementary Fig. 2.

Morphological, histological, and gene expression analyses of the hips of DA-DDH rats.

(a) Hematoxylin-eosin (HE) and safranin O/fast green (SO) staining of the hip of 10-day-old rats. Scale bar, 200 μm.

(b) Relative mRNA expression of *Col2a1*, *Acan*, *Col10a1*, *Mmp13*, and *Col1a1* in the acetabulum of 10-day-old rats (n = 8 per each group).

(c) *In situ* hybridization showing *Col2a1* and *Col1a1* expression in the acetabulum of 10-day-old rats. Scale bar, 200 μm.

(d) Second harmonic generation (SHG) analysis of the acetabular cartilage of 10-day-old rats. Scale bar, 100 μm.

(e) Terminal deoxynucleotidyl transferase-mediated dUTP nick end labeling (TUNEL) assay staining in the acetabulum of the 10-day- and 4-week-old rats. Scale bar, 100 μm.

(f) Immunostaining of Ki67 in the acetabulum of the 10-day- and 4-week-old rats. Scale bar, 50 μm. The graph shows the percentage of Ki67-positive cells in the acetabulum of the 10-day-old rats. Sections were analyzed by two observers blinded to the experiments (rats, n = 4; sections, n = 3; fields, n = 12; magnification, 20 x). White dotted lines indicate the joint line of the acetabulum (D, E, F). Mann-Whitney U test was used for statistical analysis. Values indicate the mean ± SD. * $P < 0.05$, ** $P < 0.01$, *** $P < 0.001$.

Supplementary Fig. 3.

Catabolic gene expression in the acetabular cartilage of the 10-day-old rats.

(a) Relative mRNA expression of *IL-1β*, *TNFα,* and *Nos2* in the acetabular cartilage of 10-day-old rats (n = 8 per each group).

(b) HE stainig and immunofluorescence (IF) co-staining of the acetabulum of 4-week-old rats. The dotted squares in the left panel images of each sample indicates the regions of the middle and right panels. The white line indicates the joint line of the

acetabulum, and the white dotted line shows positive region. Scale bar: HE; 100μm, IF co-staining; 50μm. Mann-Whitney U test was used for statistical analysis. Values indicate the mean ± SD. * $P < 0.05$, ** $P < 0.01$, *** $P < 0.001$.

Supplementary Fig. 4.

Gene expression and western blot analysis of chondrocytes cultured in the microgravity environment (MG).

(a) The random positioning machine was located in the incubator.

(b) Western blot for phospho-p38 (p-p38), p38, phospho-Erk1/2 (p-Erk1/2), Erk1/2, and β-catenin expression in chondrocytes cultured for 3 h in 1G and MG. β-actin was used as an internal control.

Supplementary Fig. 5. rIL-6-induced upregulated expression of Mmp3 was suppressed in primary chondrocytes of *Postn*$^{-/-}$ mice

Real-time qPCR results for Mmp3 expression in WT and Postn-/- mice primary chondrocytes treated with rIL-6 (50 ng/ml) for 24 h (n = 4 per group). Kruskal Wallis test was used for multiple comparisons. Mann-Whitney U test was used for comparisons between two groups. Values indicate the mean ± SD. * $P < 0.05$, ** $P < 0.01$, ***

$P < 0.001$.

Supplementary Fig. 6. Establishment and histological analysis of the wild-type (WT) and *Postn*$^{-/-}$ DA-DDH mice.

(a) Flow chart for mice *in vivo* experiments. Two-week-old mice were randomly devided into two groups. Mice in DA-DDH group were swaddled for 2 weeks.

(b) Macroscopic images of the acetabulum and the femoral heads of 8-week-old mice. In DA-DDH mice, the arrow indicates the hypoplastic anterior wall, and the asterisk indicates the rough articular surface of the femoral head.

(c) Safranin O/fast green (SO) staining of the anterior walls and posterior walls of the acetabulum in 8-week-old WT control and DA-DDH mice. Scale bar, 100 μm.

Supplementary Fig. 7. Histological analysis of the wild-type (WT) and *Postn*$^{-/-}$ DA-DDH mice.

(a) SO staining of the anterior wall of the acetabulum in the 6-week-old mice (n=4; WT-DA-DDH, n=4; *Postn*$^{-/-}$ DA-DDH). Scale bar: 100 μm

(b) Immunostaining (Col2a1 and Col1a1) of the anterior wall of the acetabulum in the 6-week-old mice. Scale bar: 50 μm.

(c) The percentage of IL-6 or Mmp3 positive cells in the anterior wall of the acetabulum of 6-week-old mice. Sections were analyzed by two blinded observers (mice, n = 5; sections, n = 2; fields, n = 10; magnification, 20 ×).

(d) The percentage of p-STAT3 positive cells. Sections were analyzed by two observers blinded to the experiment (mice, n = 3; sections, n = 2; fields, n = 6; magnification, 20 ×). Kruskal Wallis test was used for multiple comparisons. Mann-Whitney U test was used for comparisons between two groups. Values indicate the mean ± SD. * $P < 0.05$, ** $P < 0.01$, *** $P < 0.001$.

Supplementary Fig. 8.

(a) *In situ* hybridization images for *Postn* to support Fig. 3(b) and 3(c).

(b) Immunostaining images for Postn to support Fig. 3(b) and 3(c).

(c) *In situ* hybridization images for *Postn* to support Fig. 7(a).

(d) Immunostaining images for Postn to support Fig. 7(a).

Supplementary Fig. 9.

Uncropped gel images for p-FAK, FAK, p-Src, Src, p-p65, p65, β-catenin, and β-actin to support Fig. 4(b).

Supplementary Fig. 10.

Uncropped gel images for p-FAK, FAK, p-Src, Src, p-p65, p65, and β-actin to support Fig. 4(c).

Supplementary Fig. 11.

Uncropped gel images for Postn to support Fig. 5(b); p-STAT3, STAT3, p-p65, p65, and β-actin to support Fig. 5(c).

Supplementary Fig. 12.

Uncropped gel images for p-p38, p38, p-Erk1/2, Erk1/2, and β-catenin to support Supplementary Fig. 4(b).

Supplementary Fig. 13.

Uncropped gel images for p-STAT3, STAT3, p-p65, p65, and β-actin to support Fig. 6(a).

Supplementary Table 1.

Animals, reagents, and resources used in this study.

Supplementary Table 2.

Numbers of animals used in this study.

Supplementary Table 3.

Sequences of the qPCR primers used in this study.

Supplementary Figure 1

a

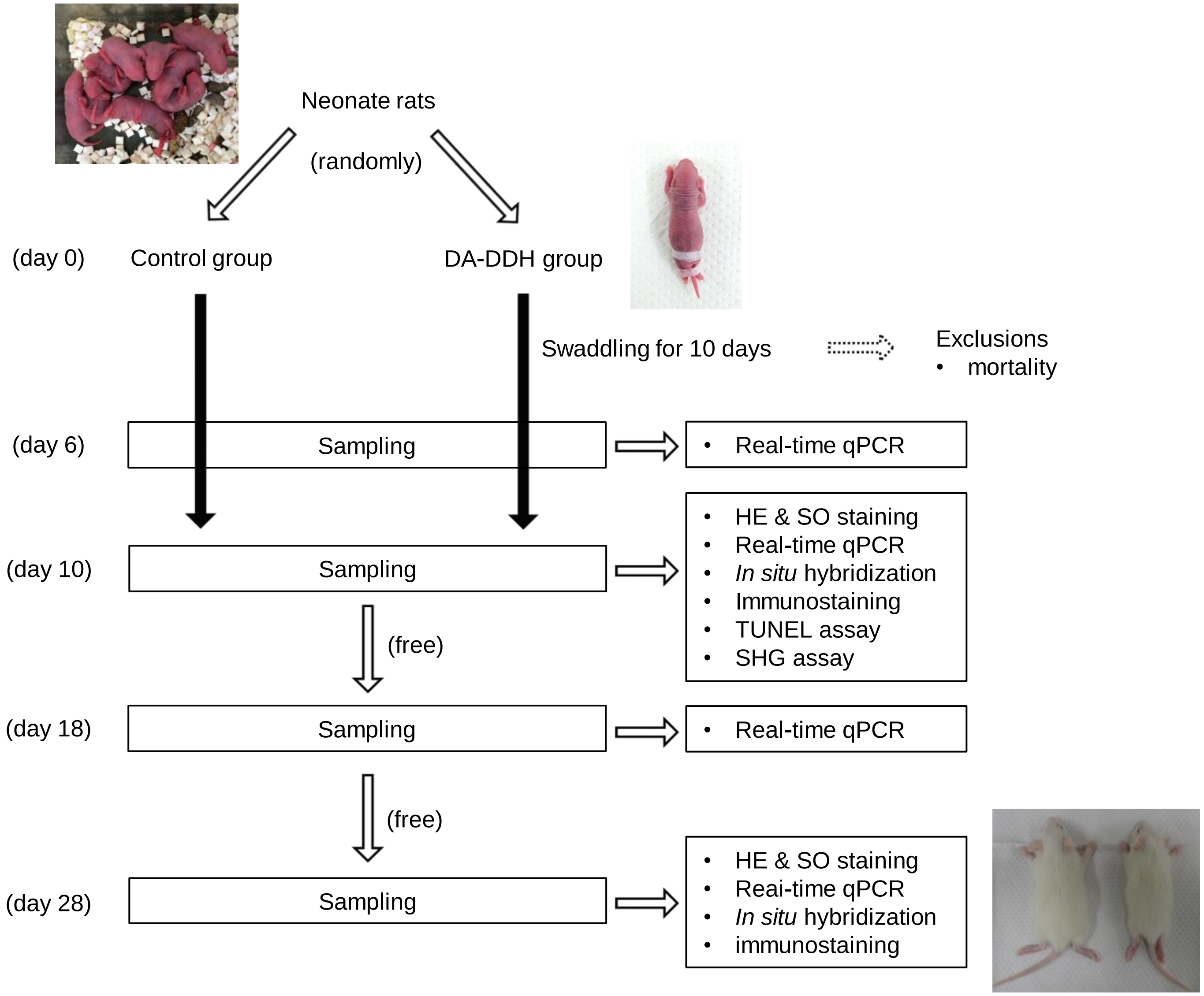

b

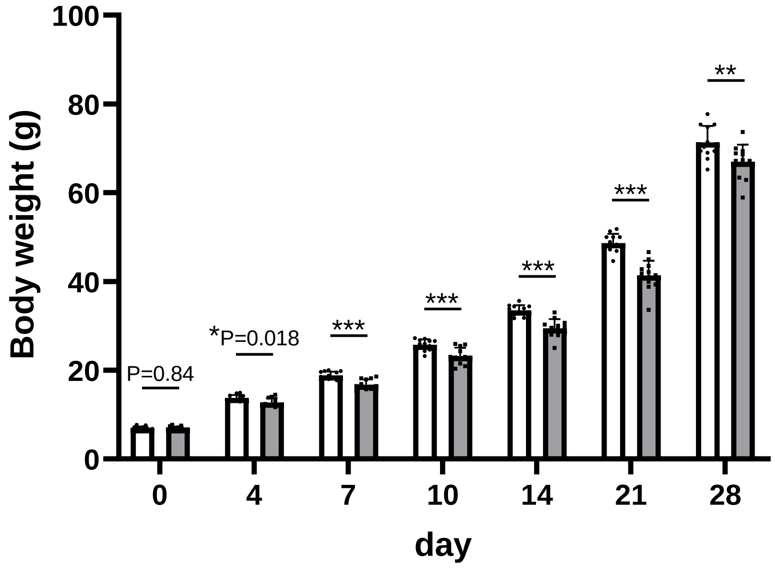

Supplementary Figure 2

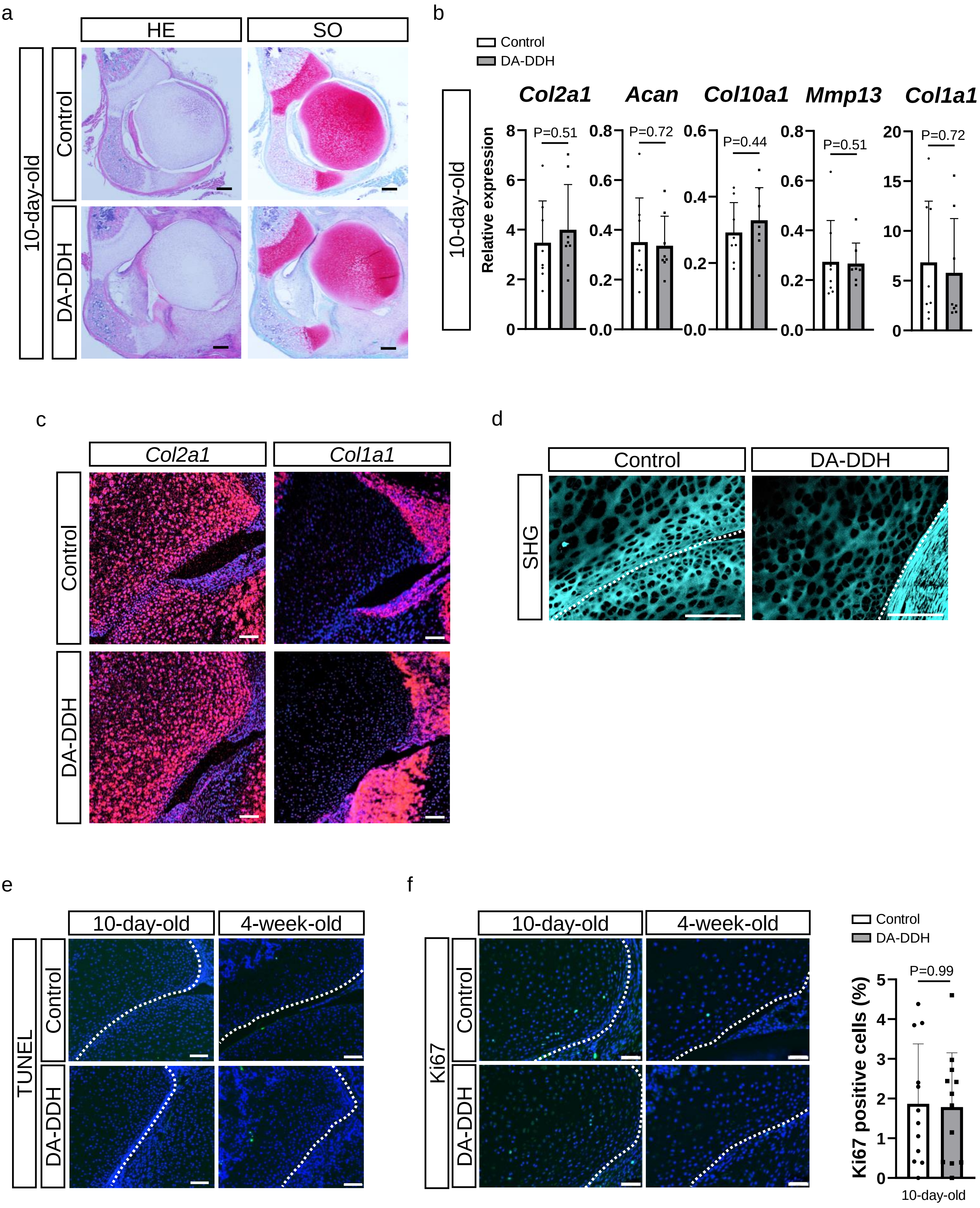

Supplementary Figure 3

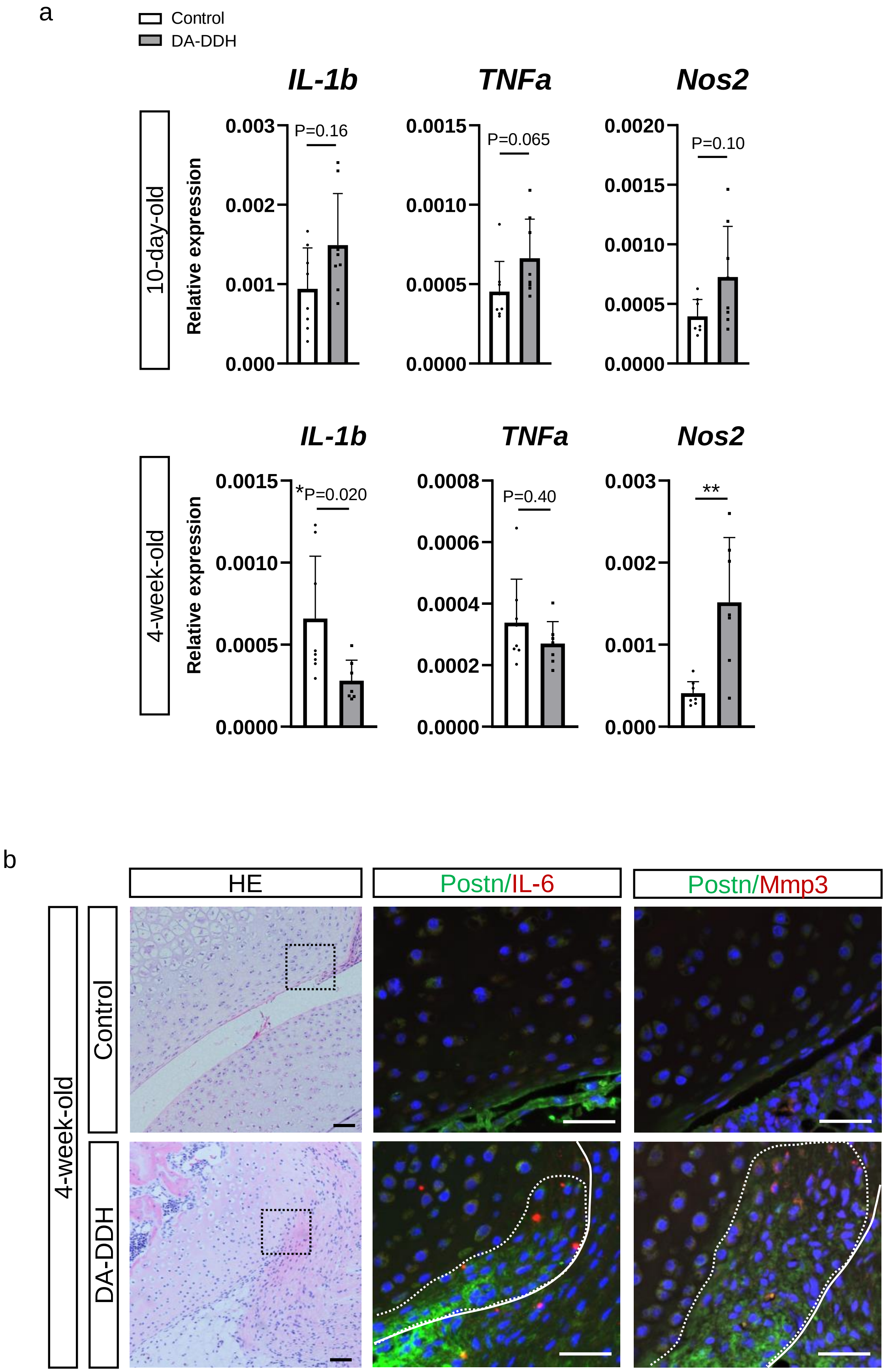

Supplementary Figure 4

a

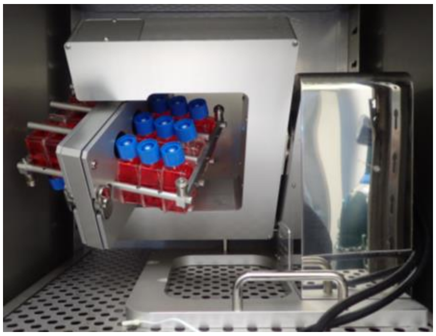

b

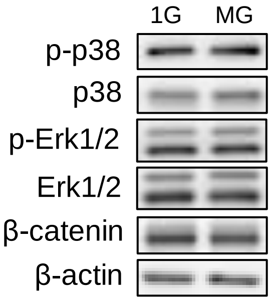

Supplementary Figure 5

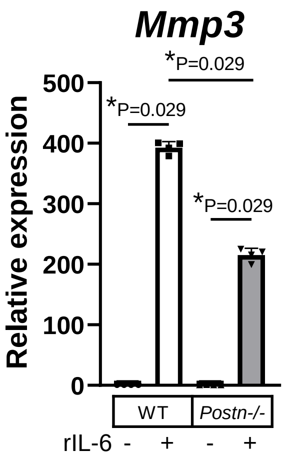

Supplementary Figure 6

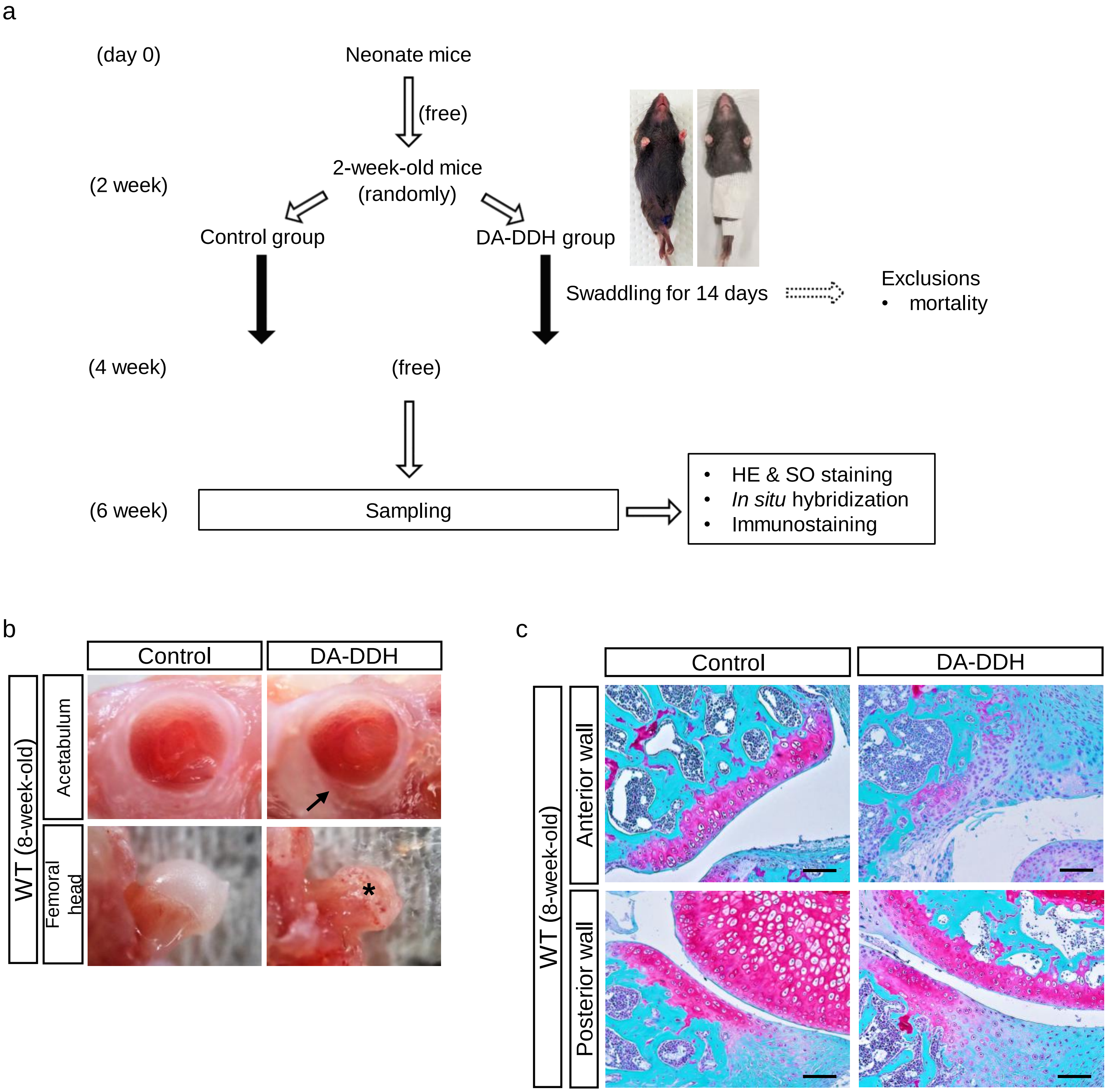

Supplementary Figure 7

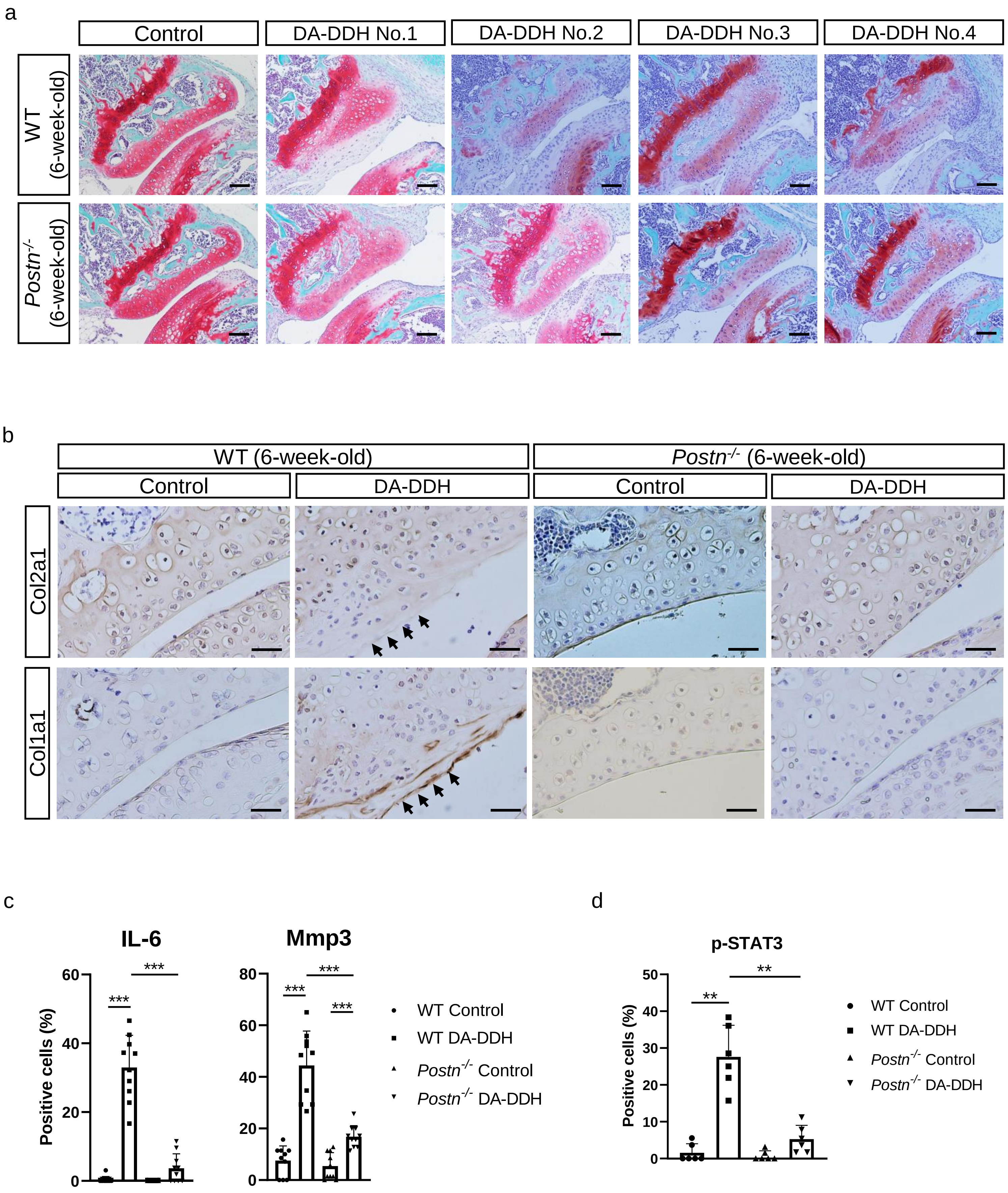

Supplementary Figure 8

a

Fig. 3b and 3c

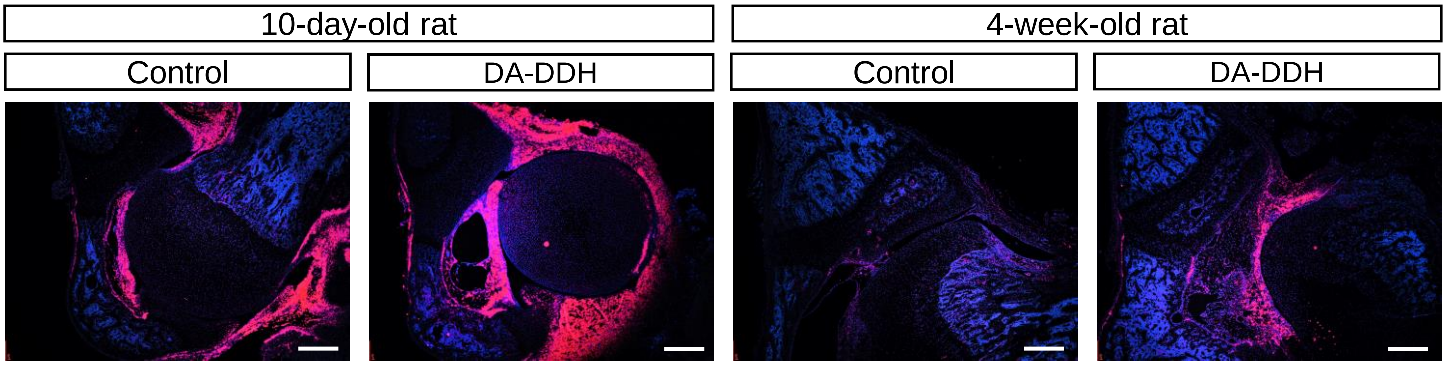

b

Fig. 3b and 3c

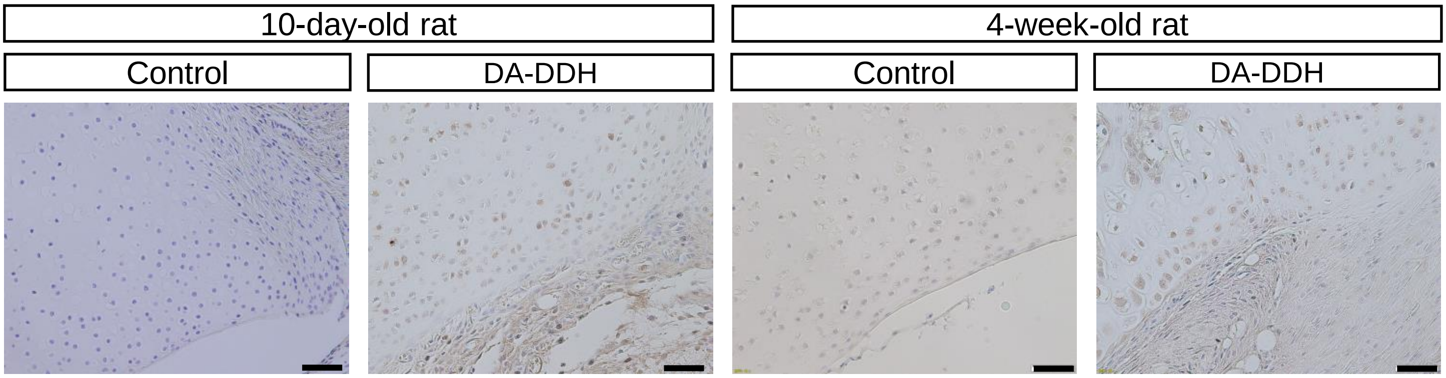

c

Fig. 7a

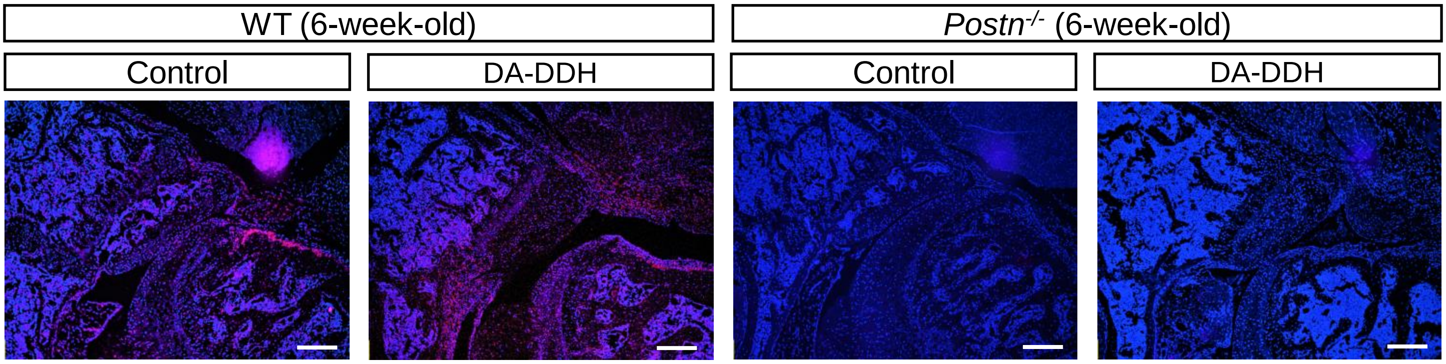

d

Fig. 7a

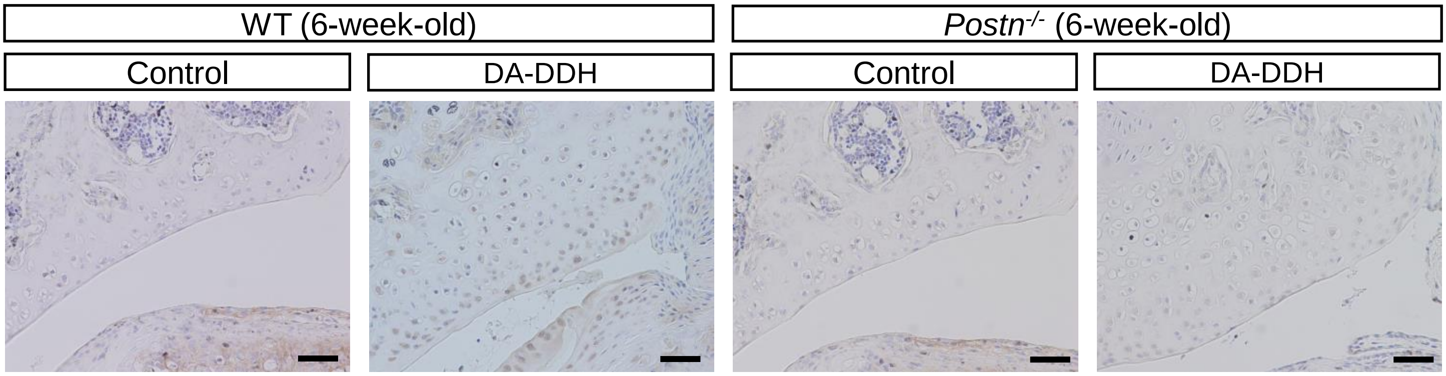

Fig. 4b

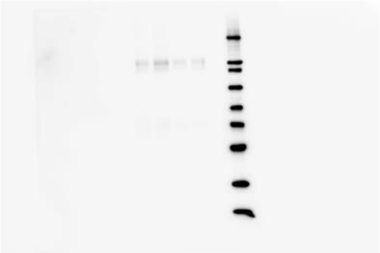

p-FAK

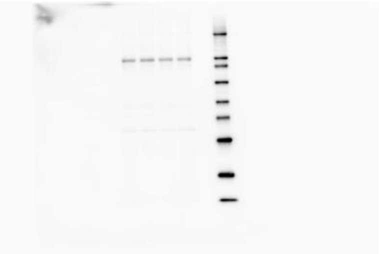

FAK

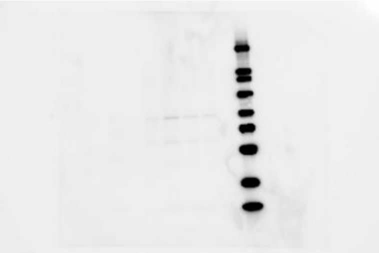

p-Src

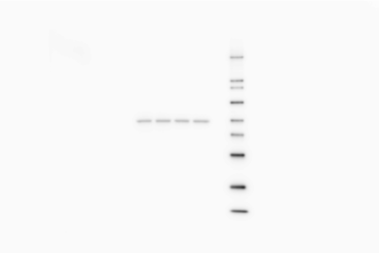

Src

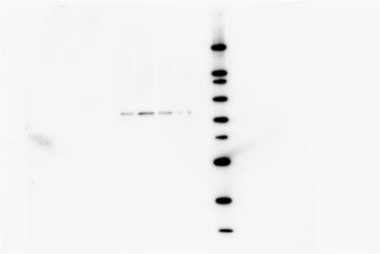

p-p65

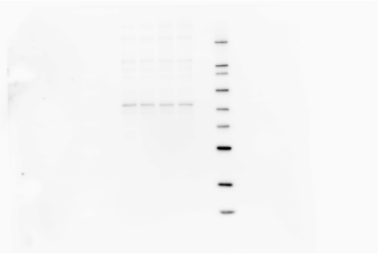

p65

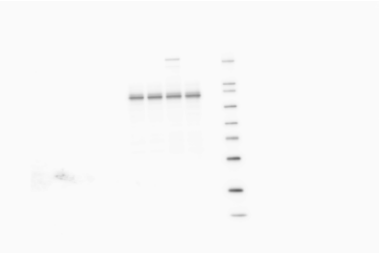

β-catenin

β-actin

Fig. 4c

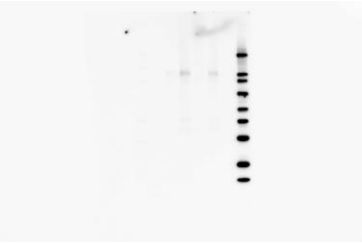

p-FAK

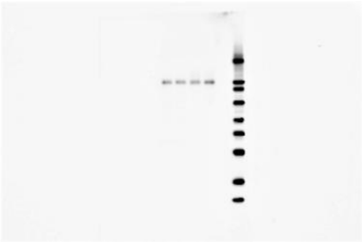

FAK

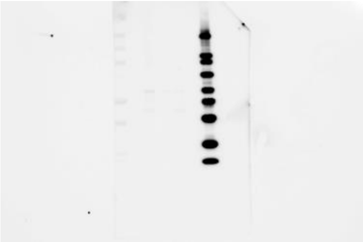

p-Src

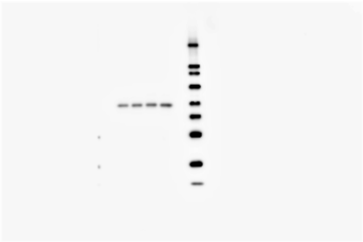

Src

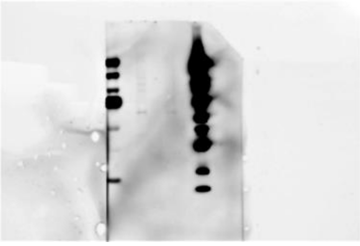

p-p65

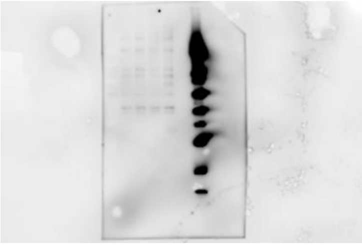

p65

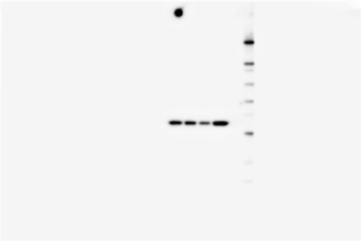

β-actin

Supplementary Figure 11

Fig. 5b

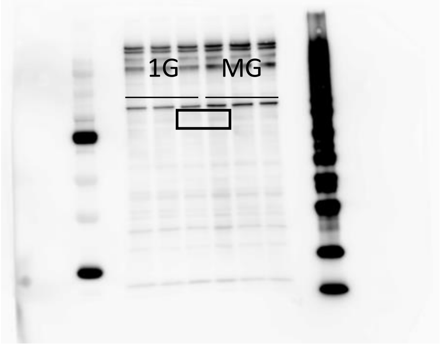

Postn

Fig. 5c

3h

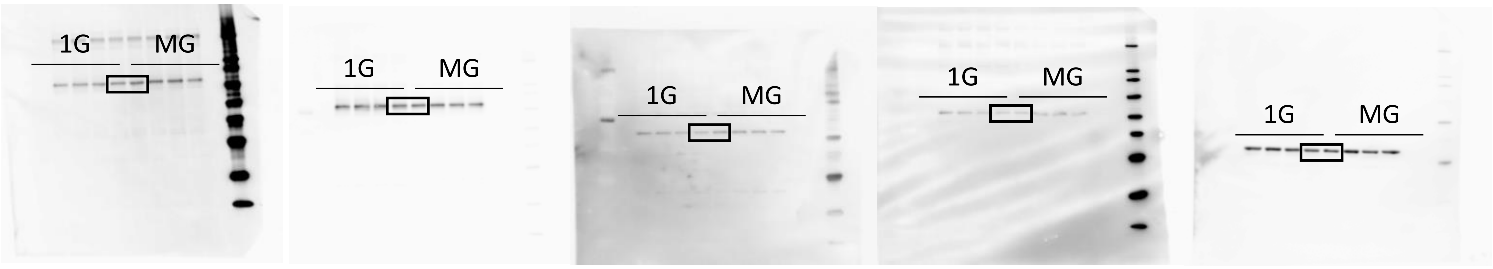

p-STAT3 STAT3 p-p65 p65 β-actin

24h

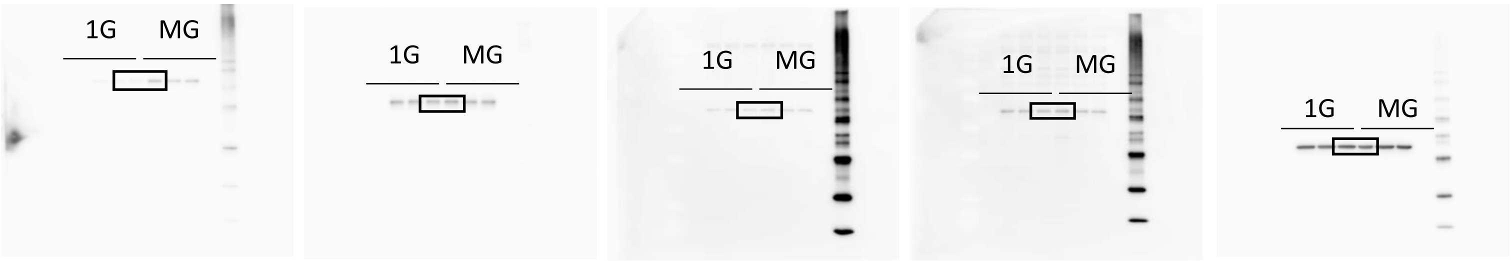

p-STAT3 STAT3 p-p65 p65 β-actin

Supplementary Fig. 4b

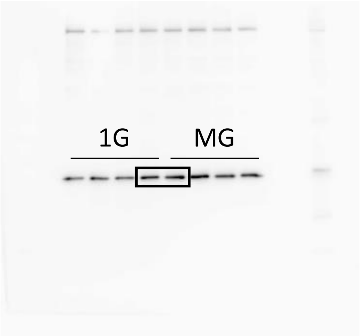

p-p38

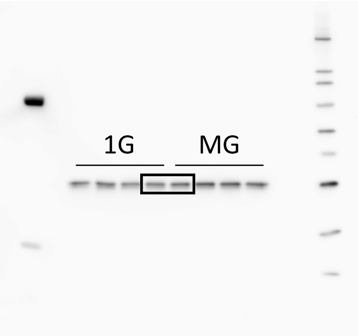

p38

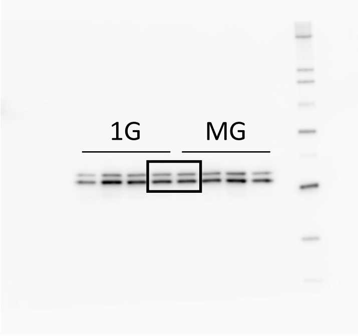

p-Erk1/2

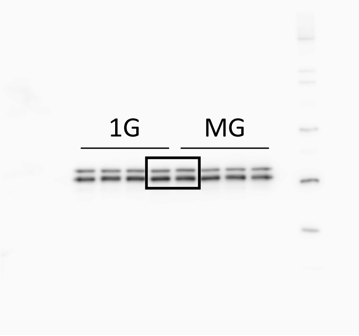

Erk1/2

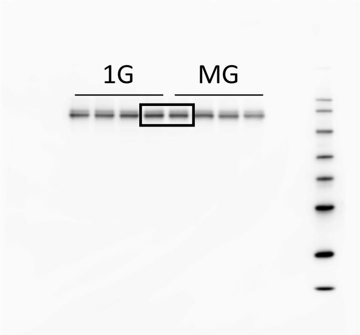

β-catenin

Supplementary Figure 13

Fig. 6a

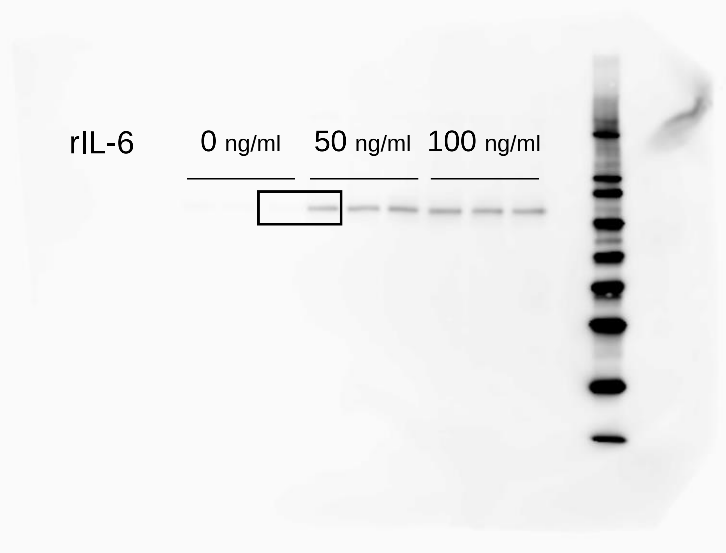

p-STAT3

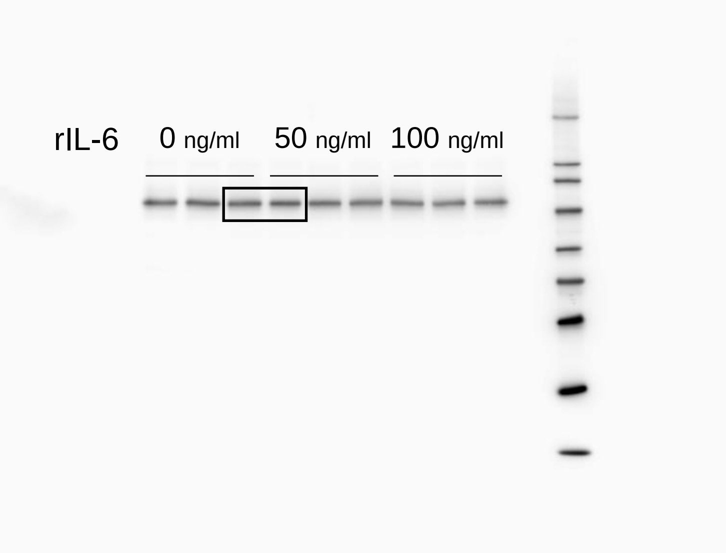

STAT3

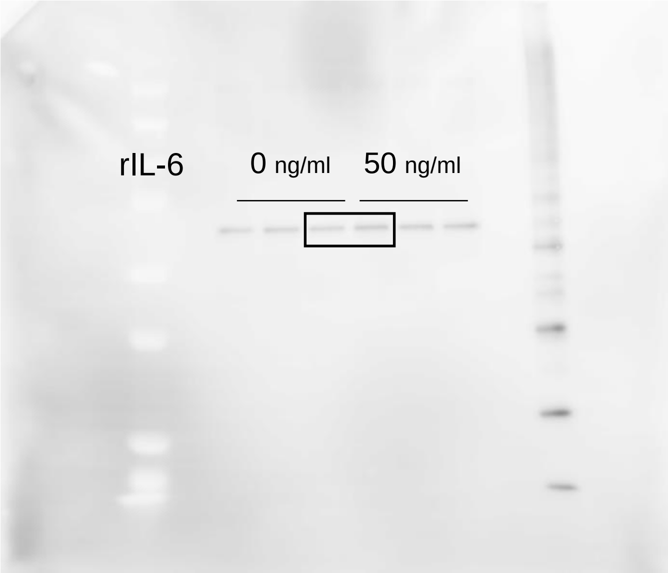

p-p65

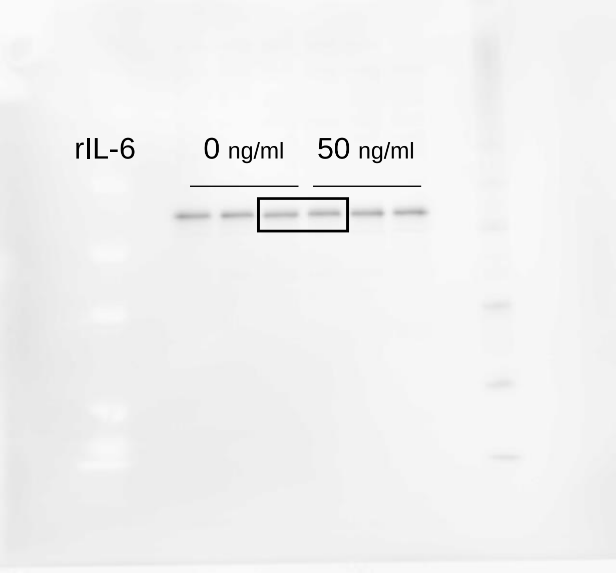

p65

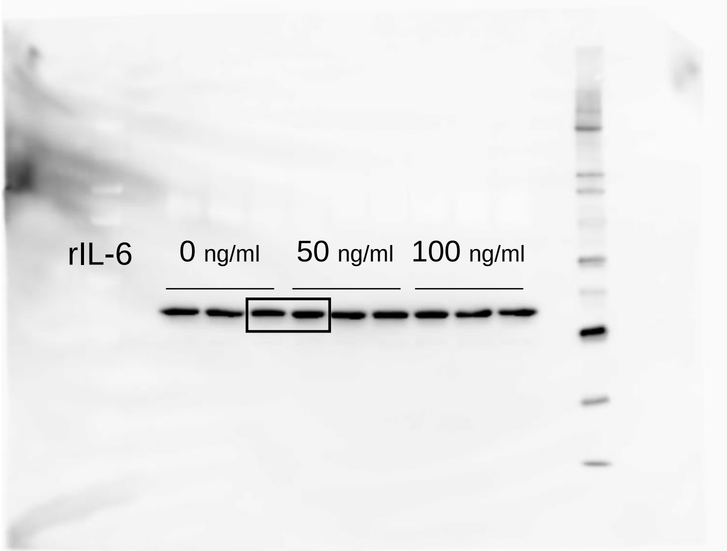

β-actin

Supplementary Table 1

Animals	SOURCE	
Wistar rat	Charles River Laboratories Japan	
C57BL/6 mouse	CLEA Japan	
Postn-knockout mouse on a C57BL/6J background	Kyushu University	
**Primary Antibodies**	**SOURCE**	**Catalog Number**
Rabbit monoclonal anti-Ki67	Abcam	Cat# ab16667
Rabbit polyclonal anti-Periostin	Abcam	Cat# ab14041
Rabbit polyclonal anti-IL-6	GeneTex	Cat# GTX110527
Rabbit monoclonal anti-MMP3	Abcam	Cat# ab52915
Rabbit polyclonal anti-Phospho FAK	Cell Signaling	Cat# 3284
Rabbit polyclonal anti-FAK	Cell Signaling	Cat# 3285
Rabbit polyclonal anti-Phospho Src	Cell Signaling	Cat# 2105
Rabbit polyclonal anti-Src	Cell Signaling	Cat# 2109
Rabbit monoclonal anti-Phospho p65	Cell Signaling	Cat# 3033
Rabbit polyclonal anti-p65	Cell Signaling	Cat# 4764
Mouse polyclonal anti-β-catenin	BD Biosciences	Cat# 610153
Rabbit polyclonal anti-β-actin	Cell Signaling	Cat# 4970
Anti-Integrin αVβ3 antibody	Millipore/Merck	Cat# MAB1876-Z
Mouse monoclonal anti-Periostin	Santa Cruz	Cat# sc-398631
Rabbit monoclonal anti-Phospho-STAT3	Cell Signaling	Cat# 9145
Mouse monoclonal anti-STAT3	Cell Signaling	Cat# 9139
Rabbit polyclonal anti-Phospho-p38	Cell Signaling	Cat# 4631
Rabbit polyclonal anti-p38	Cell Signaling	Cat# 8690
Rabbit polyclonal anti-Phospho-ERK1/2	Cell Signaling	Cat# 4376
Rabbit polyclonal anti-ERK1/2	Cell Signaling	Cat# 4695
**Secondary Antibodies, etc**	**SOURCE**	**Catalog Number**
EnVision+ System- HRP Labelled Polymer Anti-Rabbit	Dako	Cat# K4003
Liquid DAB+ Substrate Chromogen System	Dako	Cat# K3468
Donkey anti-rabbit IgG (H+L) Secondary Antibody, Alexa Fluor488	Invitrogen	Cat# A21206
Donkey anti-mouse IgG (H+L) Secondary Antibody, Alexa Fluor488	Invitrogen	Cat# A21202
Donkey anti-rabbit IgG (H+L) Secondary Antibody, Alexa Fluor594	Invitrogen	Cat# A21207
Anti-rabbit IgG, HRP-linked Antibody	Cell Signaling	Cat# 7074
Anti-mouse IgG, HRP-linked Antibody	Cell Signaling	Cat# 7076
**RI, etc**		
Uridine 5'-[alpha-thio]triphosphate, [35S]- (Sp isomer)	PerkinElmer	Cat# NEG039H
Hoechst 33258 dye	Sigma-Aldrich	Cat# 861405
**Chemicals and Recombinant Proteins**	**SOURCE**	**Catalog Number**
Cilengitide	Sigma-Aldrich	Cat# SML1594
FAK inhibitor 14	Cayman Chemical	Cat# 4506-66-5
PP2	Abcam	Cat# ab120308
BAY11-7082	Selleck Chemicals	Cat# S2913
Stattic	Abcam	Cat# ab120952
Recombinant Rat Periostin/OSF-2 Protein, CF	R&D Systems	Cat# 8994-F2
Recombinant Human IL-6, Animal Free	PeproTech	Cat# AF-200-06
**Critical Commercial Assays**	**SOURCE**	**Catalog Number**
In Situ Cell Death Detection Kit, AP	Roche	Cat# 11684809910

Supplementary Table 2

		Control	DA-DDH
**Rat (n=83)**			
6-day-old (n=11)	Real-time qPCR	n=5	n=6
10-day-old (n=24)	HE and SO staining	n=4	n=4
	Real-time qPCR	n=8	n=8
	In situ hybridization	n=1	n=1
	Immunostaining (Postn, IL-6, Mmp3)	n=1	n=1
	Immunostaining (Ki67)	n=4	n=4
	TUNEL assay	n=3	n=3
	SHG analysis	n=1	n=1
18-day-old (n=16)	Real-time qPCR	n=8	n=8
3-week-old (n=9)	RNA-seq analysis	n=4	n=5
4-week-old (n=23)	HE and SO staining	n=4	n=4
	Real-time qPCR	n=8	n=7
	In situ hybridization	n=1	n=1
	Immunostaining (Postn, IL-6, Mmp3)	n=1	n=1
	TUNEL assay	n=3	n=3
**Mouse (n=20)**			
6-week-old (n=20)	SO staining	WT: n=1, *Postn*$^{-/-}$: n=1	WT: n=4, *Postn*$^{-/-}$: n=4
	In situ hybridization	WT: n=1, *Postn*$^{-/-}$: n=1	WT: n=1, *Postn*$^{-/-}$: n=1
	Immunostaining (IL-6, Mmp3)	WT: n=5, *Postn*$^{-/-}$: n=5	WT: n=5, *Postn*$^{-/-}$: n=5
	Immunostaining (p-STAT3)	WT: n=3, *Postn*$^{-/-}$: n=3	WT: n=3, *Postn*$^{-/-}$: n=3

Supplementary Table 3

real-time qPCR Primer	Forward (5'→3')	Reverse (5'→3')
**Rat**		
Col2a1	ATTGTTGACATTGCACCCATGG	CTTTGGGTTCGCAATGGATTGT
Acan	CTGTCTATCTGCACGCCAACC	CCTCTTCACCACCCACTCCGA
Col10a1	CCAGGACACAATACTTCATCCCATACC	CCAGGAATGCCTTGTTCTCCTCTTAC
Mmp13	TCGCATTGTGAGAGTCATGCCAACA	TGTGGTTCCAGCCACGCATAGTCA
Col1a1	CGGAATGAAGGGACACAGAGGT	GCTCCATTTTCACCAGGACTGC
Postn	TCTGCTGCTGCTGTTCCTGT	GATGCGGCTGTGAGCTAGGA
IL-6	GTCCTTCCTACCCCAACTTCC	GGATGGTCTTGGTCCTTAGCC
Mmp3	GGGCTATCCGAGGTCATGAAG	CTTCTGGACGGTTTCAGGGAG
IL-1β	CTACCTATGTCTTGCCCGTGG	CTAGCAGGTCGTCATCATCCC
TNF-α	GGTATGAGCCCATGTACCTGG	CCGGACTCCGTGATGTCTAAG
Nos2	GGCCACCTCGGATATCTCTTG	CTGGGTCCTCTGGTCAAACTC
Gapdh	GTATGACTCTACCCACGGCAAGT	CTCCTGGAAGATGGTGATGGGTT
